# Computer aided progression detection model based on optimized deep LSTM ensemble model and the fusion of multivariate time series data

**DOI:** 10.1038/s41598-023-42796-6

**Published:** 2023-09-28

**Authors:** Hager Saleh, Eslam Amer, Tamer Abuhmed, Amjad Ali, Ala Al-Fuqaha, Shaker El-Sappagh

**Affiliations:** 1 Faculty of Computers and Artificial Intelligence, South Valley University, Hurghada, Egypt; 2 Communications and Information Technology, The Institute of Electronics, Queen’s University of Belfast, Belfast, UK; 3 Information Laboratory (InfoLab), College of Computing and Informatics, Sungkyunkwan University, Seoul, Suwon, 16419 South Korea; 4 Information and Computing Technology (ICT) Division, College of Science and Engineering (CSE), Hamad Bin Khalifa University, Doha, Qatar; 5 Faculty of Computer Science and Engineering, Galala University, Suez, 435611 Egypt; 6 Faculty of Computers and Artificial Intelligence, Benha University, Banha, 13518 Egypt

**Keywords:** Computational biology and bioinformatics, Data integration, Data mining, Data processing, Databases, Machine learning, Statistical methods

## Abstract

Alzheimer’s disease (AD) is the most common form of dementia. Early and accurate detection of AD is crucial to plan for disease modifying therapies that could prevent or delay the conversion to sever stages of the disease. As a chronic disease, patient’s multivariate time series data including neuroimaging, genetics, cognitive scores, and neuropsychological battery provides a complete profile about patient’s status. This data has been used to build machine learning and deep learning (DL) models for the early detection of the disease. However, these models still have limited performance and are not stable enough to be trusted in real medical settings. Literature shows that DL models outperform classical machine learning models, but ensemble learning has proven to achieve better results than standalone models. This study proposes a novel deep stacking framework which combines multiple DL models to accurately predict AD at an early stage. The study uses long short-term memory (LSTM) models as base models over patient’s multivariate time series data to learn the deep longitudinal features. Each base LSTM classifier has been optimized using the Bayesian optimizer using different feature sets. As a result, the final optimized ensembled model employed heterogeneous base models that are trained on heterogeneous data. The performance of the resulting ensemble model has been explored using a cohort of 685 patients from the University of Washington's National Alzheimer’s Coordinating Center dataset. Compared to the classical machine learning models and base LSTM classifiers, the proposed ensemble model achieves the highest testing results (i.e., 82.02, 82.25, 82.02, and 82.12 for accuracy, precision, recall, and F1-score, respectively). The resulting model enhances the performance of the state-of-the-art literature, and it could be used to build an accurate clinical decision support tool that can assist domain experts for AD progression detection.

## Introduction

Alzheimer’s disease (AD) dementia is a neurovegetative disease with a long prodromal stage that has almost no care. AD has become the fifth leading cause of death in the elderly^1^. In 2018, the dementia patients reached 50 million and it is expected that in 2050 one case of AD will be diagnosed after every 33 s with about one million new cases every year^2^. AD is a major disease that affects the health of the elderly and the causes of AD are mostly unknown yet, and there is almost no cure or a way to stop it. Therefore, early identification of patients at risk of developing AD is crucial to plan for disease-modifying therapies that could prevent or delay the conversion to sever stages of the disease^1^. Fortunately, the risk factors and symptoms of AD are reported as aging, genetics, etc. However, recent developments in machine learning could help to diagnose and predict AD based on the available large quantity of datasets like Alzheimer's Disease Neuroimaging Initiative (ADNI), Open Access Series of Imaging Studies (OASIS), and Australian Imaging Biomarkers and Lifestyle Study of Ageing (AIBL)^3^. No study in the literature built a stacking ensemble model for AD detection based on LSTM base classifiers and time series data, especially based on the well-known NACC dataset. In this study, we propose a novel stacking ensemble model based on a group of LSTM base classifiers to interpret time series data collected from the National Alzheimer’s Coordinating Center (NACC) NACC dataset. No study in the literature has proposed similar architecture, especially based on this dataset. Unlike the problem of disease progression modeling covered by the existing literature, this study has the following contributing points. (1) Propose a novel deep learning model to accurately predict AD based on a collection of medically relevant and cost-effective multivariate time series data. (2) Use the Bayesian optimizer technique to build an optimal deep stacking model using a heterogeneous set of LSTM base classifiers and different meta learners including SVM, LR, and RF. (3) Comprehensively analyze the results of different models using the NACC real and time series dataset. The study compares the performance of different classical ML models, single LSTM models based on different longitudinal modalities, and different architectures of the deep stacking ensembles based on heterogeneous LSTM models and heterogeneous time series modalities. The remainder of the paper is organized as follows. In “Related work” section reviews the related state-of-the-art related work on AD progression detection. In “Materials and methods” section presents the materials and methods that have been used in the study. In “Proposed AD progression detection framework” section represents the proposed model. Section "Experimental results" discusses the experimental results. In “Limitations and future directions” section discusses the limitations of the study and the future directions. Finally, the conclusion is discussed in “Conclusion” section.

## Related work

In this section we review the most related work of Machine learning (ML) in AD progression detection. These include the review of the role of single and multiple modalities, timeseries data, and ensemble modeling.

### Single modality single ML/DL model

ML algorithms are widely used in medical domain and proven their significant improvements in detecting and diagnosing different diseases, such as autism^4^, Parkinson’s disease^5^, dementia^6^, depression^7^, and stroke^8^ etc. Many studies have been done to diagnose AD and predict its progression^9^. Most AD studies are based on neuroimaging data, such as magnetic resonance imaging (MRI) and positron emission tomography (PET)^10–13^. Classical ML techniques like decision tree, random forest, support vector machine (SVM), logistic regression, and others have been also heavily used in AD domain^3, 9^. Rabeh et al.^14^ integrated the SVM and decision tree and built a classifier to determine whether a patient is suffering AD or MCI. The authors extracted the hippocampus, corpus callosum, and cortex region of interests (ROIs) from MRI images; they used three SVM classifiers, one for each ROI independently to classify subjects, and the final decision was made by combining the results of the three SVMs using a decision tree. Ferreira et al.^15^ used SVM and compared the diagnostic accuracy of MRI, PET, and Single-photon emission computed tomography (SPECT) images in detecting AD. Other studies used other modalities to predict AD. for example, Moore et al.^16^ used demographic and genetic data with random forest classifier to predict AD. As a subset of machine learning techniques, deep learning (DL) has received significant attention in the last few years and has been used widely in AD and other domains especially with neuroimaging data^17–19, 84, 85^. Farooq et al.^20^ proposed a 2D convolutional neural network classifier based on MRI images to determine if the subject is AD, mild cognitive impairment (MCI), NC, or late MCI. The study used transfer learning on GoogleNet, ResNet-18, and ResNet-152 models. Jain et al.^21^ utilized VGG-16 pretrained on ImageNet for feature extraction to detect AD using MRI images. Previous studies are mostly based on single modality. However, because AD is a complex disease marked by beta-amyloid and tau-mediated injuries in addition to brain atrophy and cognitive decline, physicians always consider heterogeneous multivariate data to take accurate and effective decisions^22^. Acquiring data from single modality did not provide sufficient information for diagnosis, but the fusion of multivariate data proved their effectiveness to predict longitudinal disease progression^23^. Different modalities provide information about the disease from different perspectives. As a result, the accuracy of the machine leaning models based multivariate data is better than that of single modality^24^.

### Multivariate baseline data and ML/DL models

The integration of heterogenous multivariate data (i.e., neuroimages, lab tests, memory tests, genetics, etc.) is expected to improve the performance of the ML models and supports the ML models to provide tailored and customized decisions^25^. The main reason for this behavior is because the ML models are based on the full profile of the patient and each modality offers different details for the AD which makes classifier more effective^26^. Multivariate data fusion techniques are (1) early fusion where all modalities are integrated in a single dataset which is utilized by a single ML model to predict AD, and (2) late fusion where every modality is utilized by a separate ML model and the decisions of all these models are combined to take the final decision^1^. The last method is called ensemble learning^27^. The combination of multiple ML algorithms is called ensemble learning. Ensemble techniques like bagging, boosting, voting, and stacking are expected to improve the algorithm performance^28^. Ensemble models are predefined models like random forest and extreme gradient boosting (XGboost), or they could be tailored models like stacking and voting^29^. Alickovic and Subasi^30^ explored the role of RF to diagnose AD using MRI images. Image features were extracted using the histogram, and these features were used as inputs for different classifiers including SVM, multilayer perceptron, k-nearest neighbor, random forest, naïve Bayes, logistic regression, and decision tree. The study discovered superior results of RF ensemble compared to other classifiers. Ortiz-Garcia et al.^31^ integrated MRI and PET image modalities to detect AD using the deep belief network. The study proposed a tailored ensemble of deep belief networks by integrating the four different voting algorithms of majority voting, weighted voting, SVM based data fusion, and deep belief network-based data fusion. The accuracy was about 90% for deep belief network and SVM based voting for classification of NC vs. AD subjects. Lee et al.^23^ developed a multivariate recurrent neural network (RNN) using different biomarkers including MRI images, demographic data, cognitive scores, and cerebrospinal fluid (CSF) biomarker to predict the progression of AD. An et al.^32^ integrated many clinical data including medical history, neuropsychiatric inventory questionnaire, geriatric depression scale, cerebrovascular disease, and Hachinski ischemic score using an ensemble of deep belief network to classify AD patients. The study utilized two sparse autoencoders at the voting layer to learn features, reduce the correlation of attributes, and diversify the base classifiers. The previous studies were based on multivariate of baseline data, especially neuroimaging data. Mirzaei and Adeli^3^ and Arafa et al.^25^ provided recent surveys of ML and DL techniques that have been used in AD diagnosis. However, AD is a chronic disease which is developed over time. Multivariate time series data analysis could improve the accuracy of ML and DL models^24, 33^.

### Multivariate time series data and ML/DL models

ML models have been used to learn time series data by extracting statistical features from the time series data as a preprocessing step. Then these learned features are used by classical ML models to detect or predict AD. El-Sappagh et al.^34^ fused a collection of 2.5 years’ time-series data including comorbidities, cognitive scores, medication history, and demographics. The resulting data were preprocessed to extract representative statistical features, and these features were learned using many classical ML models as SVM, k-nearest neighbor, logistic regression, and decision tree. In addition, random forest ensemble model has been explored. As expected, random forest achieved the best results. Random forest has been used by Ramírez et al.^35^ to detect MCI patients. In the TADPOLE grand challenge, (TADPOLE grand challenge: https://tadpole.grand-challenge.org/) Moore et al.^16^ applied the random forest technique to predict AD achieving an AUC of 0.82 and a 3-class classification accuracy of 0.73. Classical ML and ensemble algorithms have limitations to understand and extract deep features from time series data^36^. In comparison, many deep learning algorithms, such as convolutional neural networks (CNN) and RNN, have been designed to extract deep temporal features from time series data which are more representative than the statistical features^37^. For the most recent advances in DL studies in AD, readers are guided to this study^38^. In^36^, El-Sappagh et al. designed a two-stage long short-term memory (LSTM) based DL model for AD progression detection. The study was based on the early fusion of multivariate time-series data such as neuroimaging data, cognitive scores, CSF biomarkers, neuropsychological battery, and demographics. Robust hybrid deep learning models have already been successfully applied to AD progression detections^24^. Moreover, in the medical domain, it is not easy to introduce novel ML methods while physicians are asking for methods that are multi-modal with comprehensible recommendations^26^. In^24^, Abuhmed et al. proposed a deep multivariate bidirectional LSTM (BiLSTM) ensemble model based on the late fusion of five time series modalities including PET, MRI, neuropsychological battery, neuropathology, and cognitive scores. The extracted temporal features from the five BiLSTM models are again fused with features extracted from non-time series features (e.g., demographics and genetics) using feed forward neural network. El-Sappagh et al. proposed a hybrid CNN-LSTM deep learning model. In this architecture, five modalities were learned with five different CNN-LSTM hybrid models. The extracted features from different modalities are fused and used to predict AD progression. DL models outperformed all classical models in most AD studies. However, even the proposed DL models made late fusion of heterogeneous features, the resulting models have limitations because they did not explore the capabilities of the ensemble algorithms such as stacking.

### Ensemble modeling and time series data

An ensemble model, also known as multiple classifier model, combines a pool of intelligent classifiers seeking to exploit the strengths of each classifier in such a way to reduce the generalization error you may get from any single model^39^. Ensemble models including bagging, boosting, voting, and stacking have attracted much research for years in different application domains including AD domain, and they achieved superior results compared to other ML and DL models^40–42, 85^. Sørensen et al.^43^, proposed a bagging ensemble of SVM base classifiers. Authors asserted that the ensemble SVM outperformed single SVM classifications. Loddo et al.,^44^ proposed a voting deep ensemble model based on the three DL models of AlexNet, ResNet101, and InceptionResNetV2 as base classifiers and average voting to combine decisions. This ensemble was based on fMRI data as input and achieved an accuracy of 98.51% in the binary case, and 98.67% in the multiclass case. Ji et al.^45^ proposed ensemble model of ResNet50, NASNet, and MobileNet for diagnosing AD. Jabason et al.^46^ proposed ensemble of DenseNet and ResNet architectures based on MRI data, and the majority voting technique was applied to make the final decision. Kang et al.^47^ proposed a majority voting-based ensemble classifier for AD diagnosis. The proposed multi model multi slice ensemble selected the top 11 coronal slices of grey matter density maps for AD versus cognitive normal; then, discriminator of a generative adversarial network, VGG16, and ResNet50 were trained with the selected slices, and the majority voting was used to merge the multi-slice decisions of each model. Zhang et al.^48^ integrated 3D-VGG classifiers with weighted majority voting approach to create an ensemble classifier. However, building decision support system based on single modality (e.g., neuroimaging data as MRI) is not sufficient in medical domain because it is not trusted, the resulting ensembles did not optimize the diversity among base classifiers, and these studies did not utilize time series data. It is worth noting that current AD ensemble-based studies tend to utilize a limited amounts of training data, feature sets, and numbers of modalities while ignoring time series data completely^16, 49^.

Selecting, optimizing, and training base classifiers is the first stage in generating ensemble classifier. We can train N different algorithms, with a single training dataset, to generate N heterogeneous classifiers. Another method is to create N different portions of data from the input data and use a single classifier with each portion. For example, Choi et al.^50^ enhanced the diversity of the deep convolutional neural network base models of their ensemble classifier based on MRI data by using multiple MRI projections with different CNN architectures. In addition, the selection of the optimal fusion weights of the CNN members was designed as a generalization loss solved using the sequential quadratic programming. The rule is to adopt an approach that maximizes the diversity of base classifiers. Stacking ensemble models support the combination of both diversity enhancement approaches by selecting different features set to be used to train different base classifiers. Stacking is the training of a meta-algorithm to combine the predictions of many other learning algorithms, i.e., base classifiers. First, base algorithms are trained using selected feature set from input data, then the meta-learner is trained to make the final prediction using all the predictions of the base models as inputs. Stacking always yields performance better than any single one of the trained base models^51^. It has been successfully used in both regression and classification tasks^52, 53^. In addition, it is a popular technique in medical^54, 55^ and non-medical^56, 57^ domains. Fang et al.^58^ improved the diversity in the proposed deep stacking ensemble model by using different input data (i.e., MRI and PET images) with different base CNN classifier architectures (i.e., GoogleNet, ResNet, and DenseNet). Next, the Adaboost classifier with single decision tree classifier has been used as the meta-learner. An et al.,^32^ proposed DELearning which is a stacking ensemble model for AD diagnosis. The study integrated the baseline features of seven groups of measures from the National Alzheimer's Coordinating Center (NACC) dataset^59^ including medical history, Hachinski ischemic score, Functional Activities Questionnaire, etc. The neural network is used as a meta classifier. However, most existing studies on AD are based on the ADNI dataset, and majority of ensemble studies are based on the baseline data of MRI modality. As previously asserted, time series data analysis with suitable DL algorithms such as LSTM has achieved improved results than studies that were based on baseline data^22, 60–64^.

## Materials and methods

In this study, we predict the AD progression based on multivariate time series data analysis. We proposed a deep LSTM stacking ensemble that can interpret the time series medical data and predict if the patient will progress to AD or not. In this section, we discuss the used dataset, the formulation of the problem, the LSTM unit, and the proposed stacked DL model architecture.

### Dataset description

The University of Washington's NACC dataset^65^ is publicly available as a longitudinal AD data aiming to facilitate researchers in the field of AD. The NACC maintains a database of participant information collected from 34 past and present National Institute on Aging-funded Alzheimer’s Disease Centers. These datasets include standardized cognitive, behavioral, and functional data for each participant based on their annual visits. In this study, we used dataset of 685 subjects (i.e., cognitively normal (CN) of 229 and AD of 456). Table 1 shows the description of the selected patients. The initial number of patients in NACC dataset was 2,409. By removing patients that had no baseline visit and no regular visits, the number of patients dropped to 882. We then selected the patients that had three to six visits to build the time series dataset, and the number of patients dropped to 685. The distribution of patients with their available visits in both categories is as follows: 2-time steps (139), 3-time steps (189), 4-time steps (149), 5-time steps (125), and 6-time steps (83). Our study is based on a time series dataset of six visits against each patient. We selected 56 medically relevant and well-known features from the NACC dataset based on five modalities including A1 (Subject Demographics, A5 (subject health history), B1 (physical characteristics), B6 (geriatric depression scale (GDS) sub scores), and B7 (functional activities questionnaire (FAQ) sub scores). Detailed information about these feature categories is provided in the Supplementary Table S1. These features are significant because they provide information about the current levels of cognitive performance of a person. For example, FAQ is used by domain experts to assess the severity of the disease and to distinguish between the different stages of AD. As can be noticed, our study is based on cost-effective time series features which are easy to collect in the hospital^66^. We did not consider any neuroimaging features. Neuroimaging data are always either limited or not available, especially in developing countries, due to their cost^34^. In addition, other features like cognitive scores can accurately predict patient status more than neuroimaging features. For example, Donnelly-Kehoe et al.^67^ concluded that the maximum accuracy achieved by using MRI features does not reach the standard of using the mini-mental state examination (MMSE) by itself.

**Table 1:** Dataset description.

Feature	CN (n = 229)	AD (n = 456)	Combined (n = 685)
Sex (male/female)	119/110	225/231	344/341
Age	73.066 ± 9.074	73.840 ± 09.430	73.581 ± 09.315
# Years education	15.205 ± 3.201	15.500 ± 03.013	15.401 ± 03.078
MMSE	27.652 ± 8.268	28.089 ± 11.584	27.943 ± 10.588
GDS	73.066 ± 2.220	73.840 ± 02.291	73.581 ± 02.267

### LSTM

**Figure 1:**
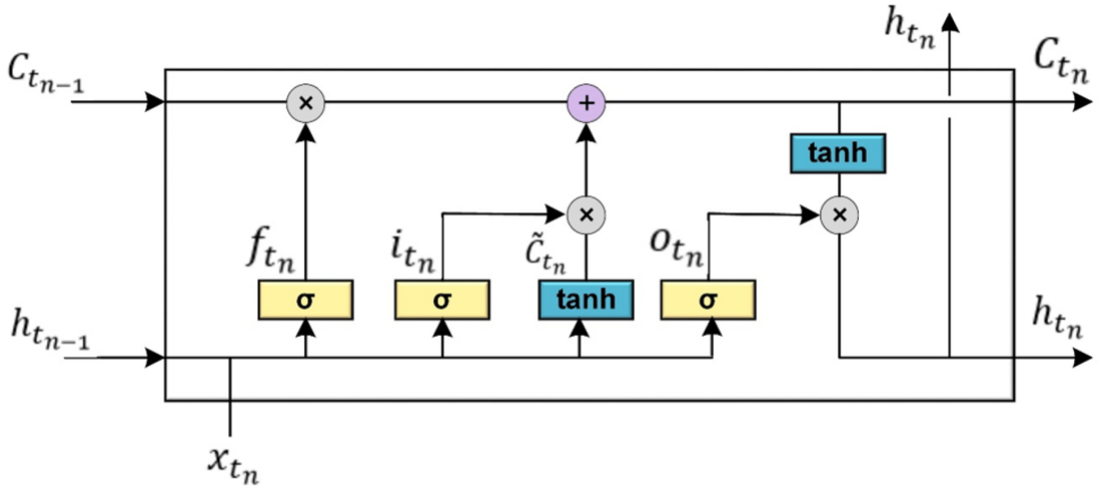
LSTM unit.

RNN are deep learning models that are naturally good at capturing longitudinal and temporal patterns in time series data. The LSTM is a new variant of the RNN that solves the problem of vanishing and exploding gradients^68^. The LSTM unit has the internal structure represented in Fig. 1. There are three gates in an LSTM cell; 1- input gate ($i_{{t_{n} }}$), 2- forget gate ($f_{{t_{n} }}$), and 3- output gate ($o_{{t_{n} }}$). The input, forget, and output gates are used to control the update, maintenance, and deletion of information contained in cell state, respectively. $C_{{t_{n} }}$,$C_{{t_{n - 1} }}$ and $\tilde{C}_{{t_{n} }}$ are the current cell status value at any time $t_{n}$, last time step cell status value, and the update of the current cell status value, respectively. $h_{{t_{n - 1} }}$ is the output value by each memory cell in the hidden layer at the previous time step. $h_{{t_{n} }}$ is the value of the hidden layer at time $t_{n}$ based on ${\tilde{\text{C}}}_{{{\text{t}}_{{\text{n}}} }}$ and ${\text{C}}_{{{\text{t}}_{{{\text{n}} - 1}} }}$. $\theta$ s and $b$ s are the set of weight matrices and biases vectors, respectively, updated following the backpropagation through time algorithm. In addition, $\otimes$ represents the Hadamard product; $\sigma$ is the standard logistic sigmoid function; $\oplus$ is the concatenation operator; $\varphi$ is the output activation function, e.g., SoftMax. The computation process of Fig. 1 is denoted as in the Eqs. (1)–(7):

1 $$f_{{t_{n} }} = \sigma \left( {\theta_{f} \cdot \left[ {h_{{t_{n - 1} }} ,x_{{t_{n} }} } \right] + b_{f} } \right)$$

2 $$i_{{t_{n} }} = \sigma \left( {\theta_{i} \cdot \left[ {h_{{t_{n - 1} }} ,x_{{t_{n} }} } \right] + b_{i} } \right)$$

3 $$\tilde{C}_{{t_{n} }} = \tanh \left( {\theta_{C} \cdot \left[ {h_{{t_{n - 1} }} ,x_{{t_{n} }} } \right] + b_{C} } \right)$$

4 $$C_{{t_{n} }} = \left( {f_{{t_{n} }} \otimes C_{{t_{n - 1} }} \oplus i_{{t_{n} }} \otimes \tilde{C}_{{t_{n} }} } \right)$$

5 $$o_{{t_{n} }} = \sigma \left( {\theta_{o} \cdot \left[ {h_{{t_{n - 1} }} ,x_{{t_{n} }} } \right] + b_{o} } \right)$$

6 $$h_{{t_{n} }} = o_{{t_{n} }} \otimes \tanh \left( {C_{{t_{n} }} } \right)$$

7 $$y_{n} = \varphi \left( {\theta_{y} h_{{t_{n} }} + b_{y} } \right)$$

LSTM-based DL architecture has been widely used for modeling sequences and time series data^22, 60, 61^. We have previously used the LSTM for diagnosing AD and predicting its progression in^24, 33, 36^.

### Stacking deep ensemble classifier

**Figure 2:**
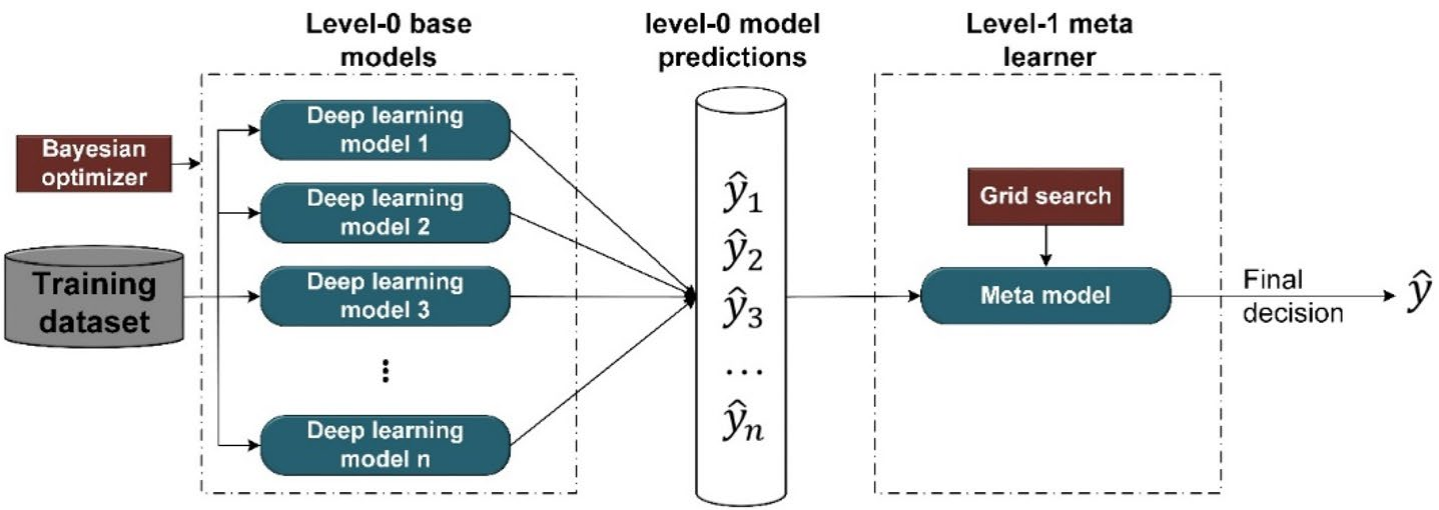
Stacking ensemble of deep learning models.

The main idea behind ensemble modeling is to weigh several base classifiers and combine their individual predictions in a way that improves the overall performance of the resulting ensemble. The key requirement for building a successful ensemble is the selection of the most accurate and diverse list of base models. This combination of these models’ predictions adds bias which in turn counters the variance of a single base model. This reduction in variance of predictions caused the ensemble to perform better than any individual best model. Stacking has the most sophisticated approach for combining the predictions of base classifiers (level-0 models). A separate ML model called meta-learner (level-1 model) is used to learn the predictions of base classifiers and automatically assigns weights to every base model based on its performance level. Meta-learner deduces the biases of base models with respect to the training sets, so meta-learner is a weighted averaging method that assigns weights to the input predictions. As a result, stacking ensemble is typically heterogeneous where its diversity comes from the different learning algorithms employed^53^. The architecture of the deep stacking ensemble model is shown in Fig. 2. To build this deep stacking ensemble model, Algorithm 1 discusses the steps of the building process. Note that the base classifiers in Algorithm 1 are deep learning (e.g., LSTM model) models with different architectures. Even the deep learning models are with the same architecture, they could be heterogeneous because each model is trained based on different number of modalities and so has different learned weights.

In Algorithm 1, the input dataset is divided into training and testing sets $D_{Train}$ and $D_{Test}$. The training set $D_{Train}$ is either used to optimize the list of base classifiers $C_{1} ,C_{2} , \ldots ,C_{P}$ or subset of its feature set is used to optimize different set of the base classifiers. The main idea is to select the best base classifier with the best list of features from $D_{Train}$. The output of the selected list of base classifiers is used to build the second level dataset that is used as input to train and optimize another meta classifier. Level 1 training set is $\left( {P + 1} \right)$-tuples: $\left\langle {cp_{1} ,cp_{2} , \ldots cp_{P} ,c} \right\rangle ,$ $cp_{i}$ is the class predicted by level 0 classifier $C_{i}$, $P$ is the number of level 0 classifiers, and $c$ is the class label. The k-fold cross validation is used to train the base classifiers, where the base classifiers are trained with k-1 folds and the predictions of the *k*^th^ fold are included in the training data set for the meta-classifier. This process is repeated *k* times which produces a new training set of the same size as $D_{Train}$. The new dataset is used to train and optimize the meta classifier. Stacking ensemble solves two issues of (1) creating out-of-sample predictions, and (2) identifying distinct regions for each model where it performs the best^69^. Based on that, the ensemble learns a different weight for each base classifier. For linear combination of base classifiers $C = \{ C_{1} ,C_{2} , \ldots ,C_{P} \}$ with weight of $W = \{ W_{1} ,W_{2} , \ldots ,W_{P} \}$, the final decision hypothesis is $h_{stacking} \left( x \right) = \sum\nolimits_{i = 1}^{P} {W_{i} C_{i} \left( x \right)}$, where the weight vector $W$ is learned by the meta-classifier, $\sum\nolimits_{i = 1}^{P} {W_{i} = 1}$, $\hat{y}_{i} = C_{i} \left( x \right)$, and $\hat{y} = h_{stacking} \left( x \right)$.

## Proposed AD progression detection framework

The proposed model is based on the NACC multivariate time series dataset. This dataset is medically divided into five different modalities. The data is randomly divided into the training/validation (80%) and the testing (20%) sets in a stratified way from the first beginning to prevent the information leakage problem. The training sets or training modalities are used to train and optimize the base classifiers independently using the k-fold cross validation technique. After that the training datasets are used to build the stacking ensemble model and select the best meta-learner. Stacking ensemble has been optimized using three meta classifiers including the SVM, the RF, and the logistic regression (LR). The optimization of models’ hyperparameters has been done using grid search, and the best meta learner has been selected. On the other hand, architectures of base LSTM classifiers are optimized using the Bayesian optimization technique to select the best hyperparameters for every base model. Different LSTM architectures are optimized for different modality combinations.
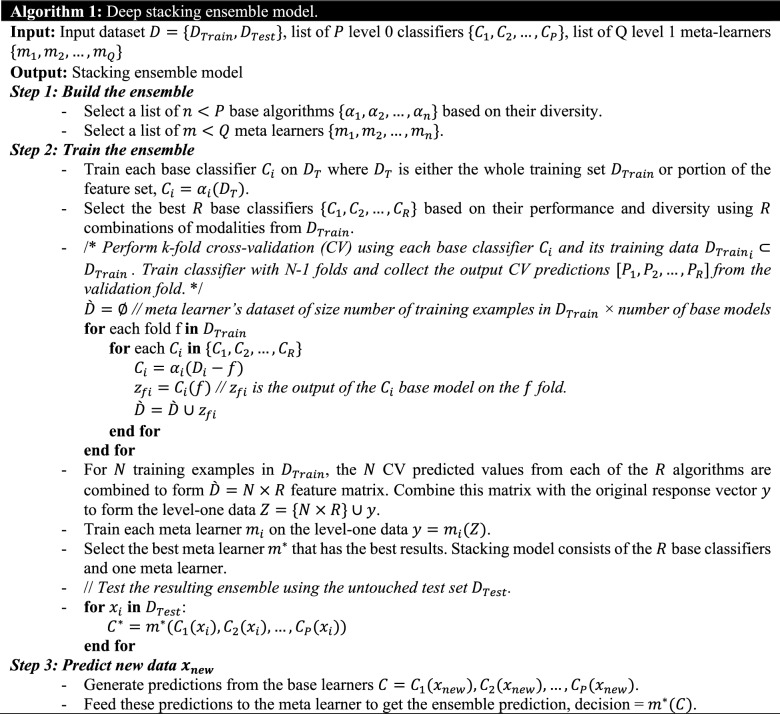


**Figure 3:**
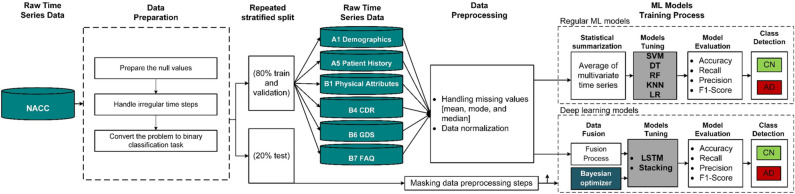
The pipeline for optimizing the base classifiers of the proposed ensemble model.

This process helps to select the best LSTM architecture and its best modality combination with different feature sets. In other words, the original dataset is divided into different subsets (i.e., modalities) with the same training examples but different feature sets. This idea is inspired by the RF technique to introduce an extra level of heterogeneity among the base classifiers. In this case, each base LSTM classifier is optimized with a different dataset, which is expected to result in a different LSTM architecture. This optimization is achieved using the Bayesian optimizer. Note that the base classifiers’ input data are based on different combinations of modalities. These combinations are medically and technically valid because combining different modalities have often been used by domain experts to make decisions and combining different modalities results in integrating heterogeneous features that complement the information provided to the classifier. In addition, selecting the best modalities is considered as a medically intuitive feature selection technique. As a result, the proposed stacking model has two sources of heterogeneity including the usage of different datasets and different base classifiers. The selection of the best number of base classifiers and the best meta-learner is based on an empirical and manual process. The general architecture of the proposed model is shown in Fig. 3. In the following subsections, we discuss each step in more details.

### Data preparation tie

Data preparation has the four sub-steps as follows:

- *Prepare the null values:* Based on the NACC documentation, it encodes missing values with different codes including 88, 888, 9, 99, 999, − 4.4, and − 4. All these values have been replaced by the NULL value and considered as missing.
- *Handle irregular time steps:* Building a time series data analysis model using deep learning depends mainly on the length of the time series. Based on the availability of visits for every patient, we selected the largest number of patients who have the highest number of visits. In this study, each patient has at most six visits. Some patients have three, four, or five visits. We regularize the number of visits by setting the values of the missing visit to zero. By using LSTM models, they will neglect these visits and consider them as if they do not exist.
- *Convert the problem to binary classification task:* The Global CDR score is a well-known clinical metric to measure the AD levels^70, 71^. This score is calculated from six cognitive sub-scores which are defined as the standard CDR scale according to clinical scoring rules^66^. Global CDR score has five different stages including 0 (no impairment), 0.5 (questionable impairment), 1 (mild impairment), 2 (moderate impairment), and 3 (severe impairment). This score has been used to determine the class of AD patient. If the value of global CDR is 0, 0.5, or 1 for all visits, the patient is considered as a cognitively normal patient. If the global CDR value is 2 or 3 in all visits or changed to one of these values during the last visits, the patient is considered a dementia case. Other researchers can utilize the proposed architecture to measure the AD progression based on other cognitive scores such as MMSE and FAQ in place of the global CDR. The resulting binary classification task has the distribution of 229 vs. 456 for Not AD vs. AD. After dividing the original modalities into 80/20 for training and testing respectively using the stratified technique, we used the oversampling technique to balance the training dataset modalities only.
- *Determine the number of visits for each patient*: based on the availability of visits data of the patients, the proposed models have been optimized based on six-time steps. The selected number of steps is sufficient to train LSTM models and minimize the missing values in the resulting dataset. The dataset is then randomly divided into 80% training set and 20% testing set using stratified methods. The training set is used to optimize the base classifiers and the stacking ensemble. The unseen test data is used to measure the generalizability of the resulting ensemble classifier.

### Data preprocessing

We adopted two preprocessing steps including the handling of the missing values and normalizing the data. Handling missing values depends on the type of data. For each patient, we replace the missing values with the mean, median, and mode values for the numerical, ordinal, or categorical data, respectively. For easier learning and fast conversion of deep learning models, each feature should have the same effect on the model performance. To achieve this goal. All numerical features have been normalized using the z-score method, i.e., $z_{j} = (x_{j} - \mu_{j} )/\sigma_{j}$ where $x_{j}$ is the participant’s original value for feature $j$, $z_{j}$ is the normalized value, $\mu_{j}$ is the feature’s mean, and $\sigma_{j}$ is the feature’s standard deviation. The z-score method converts sets of data, so they have a zero mean and unit standard deviation. The values of categorical features have been encoded. After finishing the preprocessing steps on the training dataset, these fitted operators on the training set are used to directly transform the test set. This implementation prevents the information leakage problem and allows us to test the models on untouched test data.

### Base ML models training process

The performance of the stacking ensemble model is totally based on the performance of its base classifiers and the type of feature sets used with these base models. For selecting the best base classifiers, they must be as accurate and diverse as possible. To achieve this objective, we explore many different fusion methods of multivariate time series and use each resulting dataset to optimize a different LSTM model. We use the Bayesian optimizer to select the best list of hyperparameters for each LSTM model. We tune an LSTM model with each of the six feature sets. Then, we combine two feature sets to measure the effect of adding more information on the performance of the LSTM model. Note that with the new combined feature sets, we tune a separate LSTM model. Then, we combine three feature sets and tune different LSTM models. The same process is repeated by combining four and five feature sets. The search for the best LSTM architecture is based on the Bayesian optimizer. We notice that by adding more feature sets the performance of the models is enhanced. The best LSTM model has been selected based on the fusion of B7 feature set with other feature sets. Based on the results of one modality-based LSTM models, we fused this modality with other and built other 2-feature sets LSTM models, etc.

### Stacking model training process

**Figure 4:**
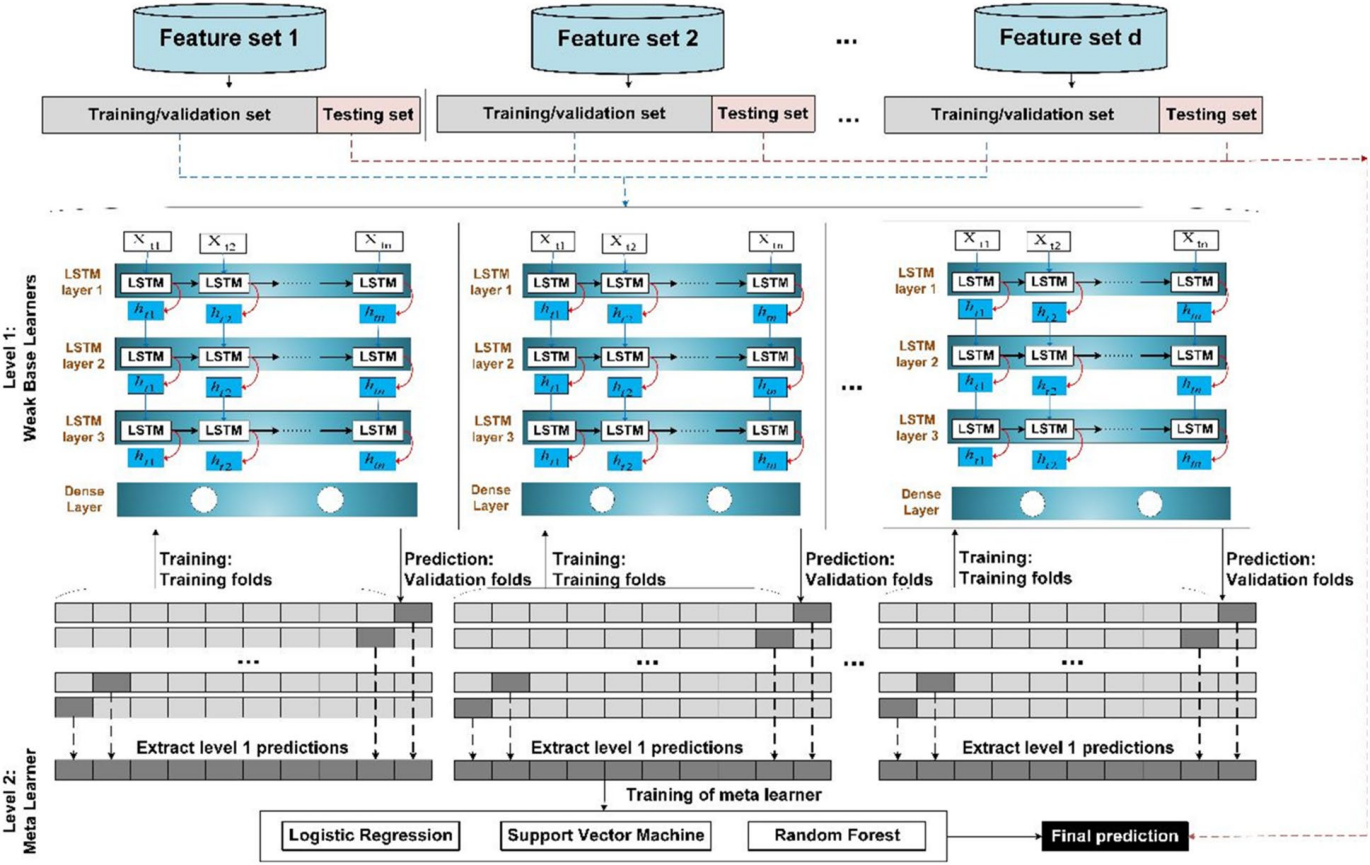
Proposed multimodal deep LSTM stacking ensemble models.

The training process for the proposed stacking ensemble is based on two stages as discussed in Algorithm 1. The first stage is to select the optimum ensemble architecture with the best number of LSTM base classifiers and the best sets of timeseries feature sets for each classifier. The second stage is to select the best meta classifier based on the outputs of the level 1 classifiers, see Fig. 4. In the following subsections, we discuss these steps in more detail.

#### Level 1 classifiers

**Figure 5:**
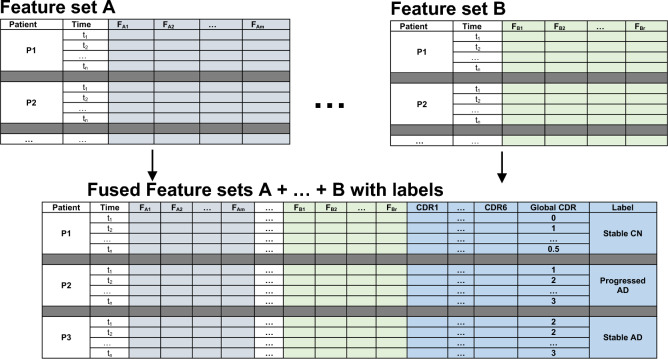
Multivariate timeseries data fusion format.

Our dataset is divided into four multivariate time series feature sets plus one static modality to learn AD progression detection problem. Each modality has a collection of features which are medically related. Each of these feature sets is either used alone to train an LSTM model or combined with other feature sets and used to optimize an LSTM model, as shown in Fig. 5. To fuse the demographics static feature set with other time series feature sets, we have repeated the same values with every time step. In our experiments, we explore the best combination of feature sets that achieve the best results. Each modality has six-time steps. We are based on early fusion mechanism of multivariate data, where data are fused and then jointly inputted to the DL model pipeline, see Fig. 5. The formulation of the classification problem is illustrated in Fig. 5 where the patient can be considered as normal, progressed AD, or AD based on the values of his/her CDR values over time. For ${\text{M}}$ feature sets of data represented as ${\text{X}} = \left\{ {{\text{X}}^{\left( 1 \right)} , \ldots ,{\text{X}}^{{\left( {\text{M}} \right)}} } \right\}$, and output ${\text{ y}}$. Each modality ${\text{X}}^{{\text{m}}}$ is represented as ${\text{X}}^{{\text{m}}} = \left\{ {{\text{x}}_{1}^{{\left( {\text{m}} \right)}} , \ldots {\text{x}}_{{\text{i}}}^{{\left( {\text{m}} \right)}} , \ldots ,{\text{x}}_{{\text{N}}}^{{\left( {\text{m}} \right)}} } \right\}$ from ${\text{N}}$ patient examples, and each example ${\text{ x}}_{{\text{i}}}^{{\left( {\text{m}} \right)}}$ is a multivariate time series ${\text{ x}}_{{\text{i}}}^{{\left( {\text{m}} \right)}} = \left\{ {{\text{x}}_{{{\text{i}}1_{t} }}^{{\left( {\text{m}} \right)}} ,{\text{ x}}_{{{\text{i}}2_{t} }}^{{\left( {\text{m}} \right)}} , \ldots ,{\text{ x}}_{{{\text{if}}_{t} }}^{{\left( {\text{m}} \right)}} } \right\}$, for $t = 1, \ldots ,s$ time-steps and $f$ set of univariate time series. For ${\text{N}}$ patients, each patient $i$ is represented as ${\text{x}}_{{\text{i}}} = \left\{ {{\text{x}}_{{\text{i}}}^{\left( 1 \right)} , \ldots {\text{x}}_{{\text{i}}}^{{\left( {\text{m}} \right)}} , \ldots ,{\text{x}}_{{\text{i}}}^{\left( M \right)} ,{\text{y}}} \right\}, {\text{i}} = 1, \ldots ,{\text{N}}$. We optimize an LSTM mode for each time series modality. In addition, extensive experiments are performed using two, three, four, and five modality fusions to select the best combinations of feature sets that achieve the best results. Binary cross-entropy cost function is used with all LSTM models, and Adam optimizer is used to search for the best weights of the neural network. The resulting LSTM models with the best modality combinations are used as base classifiers in the stacking ensemble. The best stacking architecture is based on seven LSTM base models. The best LSTM model is selected for every modality (i.e., A1, A5, B1, B6, and B7), and another LSTM base model has been optimized based on the whole feature set, see Fig. 5. The selection of base LSTM models is based on the performance of the optimized models on different feature sets combinations.

Stacking ensemble model based on seven base LSTM classifiers and SVM meta model achieved the best cross-validation results. A separate LSTM model has been optimized with every modality of A1, A5, B1, B6, and B7. In addition, an LSTM model has been optimized with the fused dataset of all features. For the single modality-based LSTM models, the Bayesian optimizer selected the best architectures as follows. The learning rate is 0.00001, the activation function is ReLu, optimizer is Adam, batch size is 50, number of epochs is 30, SoftMax is in the output layer, and categorical cross entropy is the cost function. For A1-based model, the optimized architecture has one LSTM layer (210 units), 0.3 dropout, and L2 regularizer (0.3). For A5-based model, the optimized architecture has one LSTM layer (370 units), 0.5 dropout, and L2 regularizer (0.3). For B1-based model, the optimized architecture has one LSTM layer (150 units), 0.5 dropout, and L2 regularizer (0.1). For B6-based model, the optimized architecture has one LSTM layer (210 units), 0.5 dropout, and L2 regularizer (0.01). For B7-based model, the optimized architecture has one LSTM layer (490 units), 0.3 dropout, and L2 regularizer (0.3). The best LSTM model with the fused feature sets has an optimized architecture with 3 LSTM layers (530, 330, 110), dropout of (0.2,0.5,0.2), L2 regularizer (0.05,0. 1, 0.3), epochs of 35, and batch size of 50.

#### Level 2 classifier

The $P$ base classifiers at level 1 generate $P$ outputs $\hat{y}_{1} , \hat{y}_{1} , \ldots ,\hat{y}_{P}$ based on the input multivariate time series data. The resulting $\hat{y}_{i}$ data are not time series. These output data are combined with the actual output $y$ to form a new non-time series data, which is used to optimize the meta learner. We optimize the hyperparameters of three meta learners including SVM, logistic regression, and random forest using the grid search technique. SVM achieves the best results as a meta learner. The hyperparameters of the SVM classifier have been optimized using grid search. The final hyperparameters list is C = 6, kernel = “poly”, gamma = “scale”.

## Experimental results

### Evaluation metrics

The performance of the base classifiers and ensemble models is measured in terms of accuracy, precision, recall, and F1-score, which are defined as in Eqs. (8)–(11). The TP is the true positive, the TN is the true negative, the FP is the false positive, and the FN is the false negative. These are the most used in the medical informatics literature to increase the possibilities of results comparison.

8 $$Accuracy = \frac{TP + TN}{{TP + TN + FP + FN}}$$

9 $${\text{Precision}} = \frac{TP}{{TP + FP}}$$

10 $$Recall = \frac{TP}{{TP + FN}}$$

11 $$F1 - score = \frac{2 \times Precision \times Recall}{{Precision + Recall}}.$$

### Experimental setup

To evaluate the performance of the proposed LSTM stacking model, we implement, test, and compare many DL architectures with different modality combinations. For all experiments, we employed a machine with Intel core i7-6700 CPU and 32 GB of RAM. The proposed methods are implemented by using Python 3.8 distributed in Anaconda 4.7.7 (64-bit). The proposed models are implemented using Keras library based on TensorFlow as backend. A SoftMax activation function with cross-entropy loss is used for the classification task. Adam optimizer is used with a fixed learning rate of 0.001. The training batch size and number of epochs are 30 and 50, respectively. To prevent overfitting, we use dropout, L2 regularization, and early stopping mechanisms. Regular machine learning models have been implemented using Scikit-Learn. To show the robustness of the proposed model, we compared it with other LSTM-based DL and regular ML classifiers. The dataset is divided into 80% training and validation and 20% testing. The nested cross validation has been used to validate machine learning and deep learning models. The models have been tested using untouched datasets which prevent the possibility of data leakage, and the testing results have been reported. The performance of the models is compared using the non-parametric Kruskal–Wallis statistical test. The $\alpha = 0.05$ is considered statistically significant.

### Results of regular machine learning models

**Figure 6:**
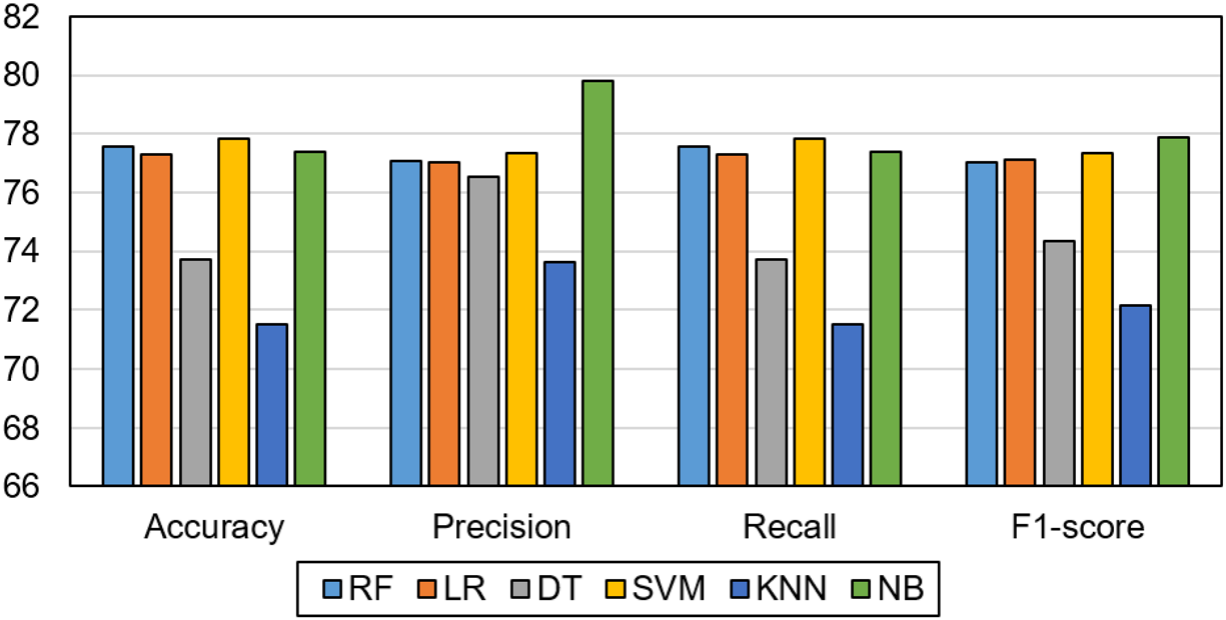
Comparison of the best performing ML model with the B7 feature set.

We evaluated our proposed framework against the regular ML classifiers, such as the decision tree (DT), the K-nearest neighbor (KNN), the LR, the SVM, and the RF. The performance of these regular ML models is considered as the base line performance. To formulate the classification task, the last visit of the patient’s time series data is used as the input to the ML models, and the output is the same as in deep learning models. We assessed and analyzed our framework performance concerning the given features in our evaluation. The results are shown in Table 2. We evaluated models’ performance using single modalities and different combinations of modalities. Because classical ML models are simple, they have not benefitted from different fusions. B7 modality achieved the best testing results with RF (i.e., 77.56, 77.09, 77.56, and 77.02 for accuracy, precision, recall, and F1-score, respectively), and A1 achieved the worst performance with RF (i.e., 60.58, 55.90, 60.58, and 57.06 for accuracy, precision, recall, and F1-score, respectively). B7 modality achieved the best testing results with LR classifier (i.e., 77.29, 77.03, 77.29, and 77.12 for accuracy, precision, recall, and F1-score, respectively), and A5 achieved the worst performance (i.e., 64.23, 57.37, 64.23, 56.98 for accuracy, precision, recall, and F1-score, respectively). B7 achieved the best results with DT classifier (i.e., 73.72, 76.52, 73.72, and 74.36 for accuracy, precision, recall, and F1-score, respectively); however, A5 achieved the worst results (i.e., 55.47, 56.65, 55.47, and 55.99 for accuracy, precision, recall, and F1-score, respectively). With SVM, B7 achieved the best results (i.e., 77.83, 77.33, 77.83, and 77.35 for accuracy, precision, recall, and F1-score, respectively), and B6 achieved the worst results (i.e., 63.50, 57.06, 63.50, and 57.26 for accuracy, precision, recall, and F1-score, respectively). B7 had the best results with KNN (i.e., 71.53, 73.65, 71.53, and 72.14 for accuracy, precision, recall, and F1-score, respectively), and A5 had the worst results (i.e., 59.12, 51.54, 59.12, and 53.63 for accuracy, precision, recall, and F1-score, respectively). With NB classifier, again B7 achieved the best results (i.e., 77.37, 79.82, 77.37, and 77.90 for accuracy, precision, recall, and F1-score, respectively), but A1 achieved the worst results (i.e., 51.09, 47.74, 51.09, and 49.17 for accuracy, precision, recall, and F1-score, respectively). The best performing modality was B7. SVM is the best classifier, and KNN was the worst one. As a result, B7 is used for optimizing the data fusion process, where we gradually fuse it with other feature sets, as discussed in the next experiment. Figure 6 shows a comparison among different regular ML models using the B7 dataset. We observed no significant difference between RF and LR, but RF is significantly different from other ML models (*P*-value = 0.03). The regular ML models are not good in learning time series data. Deep learning models like LSTM can learn the temporal patterns in longitudinal data collected over time for chronic diseases like AD. In the next experiment, we experimented with different feature sets using the LSTM model. In these experiments, we evaluated the performance of individual feature sets and different combinations of feature sets. We aimed at determining the best fusion of feature sets which enhances the accuracy of the resulting model.

**Table 2:** Performance of regular ML techniques with the last visit data.

Models	Feature set	Accuracy	Precision	Recall	F1-score
RF	A1	60.58 ± 0.93	55.90 ± 1.65	60.58 ± 0.93	57.06 ± 0.93
	A5	62.04 ± 3.06	57.79 ± 3.65	62.04 ± 3.06	58.65 ± 2.79
	B1	67.88 ± 1.75	65.21 ± 2.75	67.88 ± 1.75	64.57 ± 1.96
	B6	67.15 ± 1.78	63.72 ± 1.6	67.15 ± 1.78	61.85 ± 1.4
	**B7**	**77.56 ± 2.52**	**77.09 ± 2.72**	**77.56 ± 2.52**	**77.02 ± 2.2**
	B7A1	64.07 ± 1.58	60.24 ± 2.27	64.07 ± 1.58	60.53 ± 1.95
	B7A5	65.00 ± 1.70	59.94 ± 3.86	65.00 ± 1.70	59.56 ± 3.54
	B7B1	62.91 ± 2.25	57.03 ± 3.76	62.91 ± 2.25	57.49 ± 2.72
	B7B6	64.3 ± 1.67	59.07 ± 3.08	64.3 ± 1.67	58.73 ± 2.32
	B7A1A5	63.72 ± 1.79	57.67 ± 3.15	63.72 ± 1.79	57.45 ± 1.84
	B7A1B1	66.51 ± 3.58	63.23 ± 5.69	66.51 ± 3.58	61.77 ± 3.7
	B7A1B6	63.02 ± 1.86	58.19 ± 2.64	63.02 ± 1.86	58.61 ± 1.98
	B7A5B1	64.19 ± 2.03	59.39 ± 3.48	64.19 ± 2.03	59.15 ± 2.54
	B7A5B6	64.53 ± 1.87	58.26 ± 3.71	64.53 ± 1.87	57.62 ± 2.61
	B7B1B6	65.7 ± 1.84	61.98 ± 2.9	65.7 ± 1.84	61.25 ± 2.5
LR	A1	66.42 ± 0.28	53.12 ± 0.06	66.42 ± 0.28	53.02 ± 0.14
	A5	64.23 ± 3.42	57.37 ± 5.08	64.23 ± 3.42	56.98 ± 4.2
	B1	64.96 ± 1.40	58.63 ± 8.81	64.96 ± 1.4	57.45 ± 4.18
	B6	67.15 ± 1.47	63.64 ± 2.49	67.15 ± 1.47	61.20 ± 0.94
	**B7**	**77.29 ± 0.73**	**77.03 ± 5.68**	**77.29 ± 0.73**	**77.12 ± 2.62**
	B7A1	63.60 ± 1.50	58.72 ± 2.11	63.60 ± 1.50	58.57 ± 1.23
	B7A5	61.98 ± 2.07	56.76 ± 2.49	61.98 ± 2.07	57.27 ± 1.45
	B7B1	60.98 ± 2.07	56.76 ± 2.49	61.98 ± 2.07	57.27 ± 1.45
	B7B6	61.98 ± 2.07	56.76 ± 2.49	61.98 ± 2.07	57.27 ± 1.45
	B7A1A5	64.54 ± 1.16	60.06 ± 1.56	64.54 ± 1.16	59.83 ± 1.51
	B7A1B1	65.58 ± 0.93	61.5 ± 1.62	65.58 ± 0.93	60.74 ± 1.24
	B7A1B6	63.6 ± 0.46	59.09 ± 2.27	63.6 ± 0.46	59.58 ± 2.33
	B7A5B1	61.98 ± 2.07	56.76 ± 2.49	61.98 ± 2.07	57.27 ± 1.45
	B7A5B6	63.72 ± 0.79	59.37 ± 0.65	63.72 ± 0.79	59.55 ± 1.03
	B7B1B6	62.67 ± 0.68	58.56 ± 0.55	62.67 ± 0.68	59.14 ± 0.66
DT	A1	64.96 ± 2.36	61.90 ± 1.03	64.96 ± 2.36	62.28 ± 1.5
	A5	55.47 ± 1.74	56.65 ± 2.26	55.47 ± 1.74	55.99 ± 1.82
	B1	56.93 ± 1.64	56.23 ± 1.03	56.93 ± 1.64	56.56 ± 1.25
	B6	62.77 ± 2.25	61.79 ± 2.45	62.77 ± 2.25	62.20 ± 2.3
	**B7**	**73.72 ± 1.64**	**76.52 ± 1.64**	**73.72 ± 1.64**	**74.36 ± 1.63**
	B7A1	58.49 ± 3.22	58.96 ± 2.78	58.49 ± 3.22	58.69 ± 3.03
	B7A5	59.19 ± 2.36	58.71 ± 2.04	59.19 ± 2.36	58.85 ± 2.11
	B7B1	58.02 ± 1.44	57.69 ± 1.85	58.02 ± 1.44	57.8 ± 1.55
	B7B6	57.91 ± 2.07	57.18 ± 2.25	57.91 ± 2.07	57.49 ± 2.14
	B7A1A5	57.09 ± 1.58	55.94 ± 1.5	57.09 ± 1.58	56.43 ± 1.49
	B7A1B1	57.21 ± 1.5	57.07 ± 1.26	57.21 ± 1.5	57.09 ± 1.3
	B7A1B6	57.56 ± 1.73	58.95 ± 1.95	57.56 ± 1.73	58.1 ± 1.75
	B7A5B1	58.02 ± 2.51	57.53 ± 3.06	58.02 ± 2.51	57.71 ± 2.72
	B7A5B6	57.91 ± 2.95	58.72 ± 2.34	57.91 ± 2.95	58.23 ± 2.69
	B7B1B6	59.42 ± 1.44	59.04 ± 1.75	59.42 ± 1.44	59.16 ± 1.49
SVM	A1	66.42 ± 0.46	53.12 ± 4.13	66.42 ± 0.46	53.02 ± 0.39
	A5	64.96 ± 2.43	59.21 ± 4.96	64.96 ± 2.43	58.24 ± 2.66
	B1	65.69 ± 0.46	61.50 ± 3.75	65.69 ± 0.46	60.78 ± 1.91
	B6	63.50 ± 3.04	57.06 ± 5.96	63.50 ± 3.04	57.26 ± 3.07
	**B7**	**77.83 ± 1.63**	**77.33 ± 1.63**	**77.83 ± 1.63**	**77.35 ± 1.63**
	B7A1	63.02 ± 0.46	51.86 ± 2.25	63.02 ± 0.46	53.77 ± 0.93
	B7A5	64.07 ± 2.03	52.38 ± 3.20	64.07 ± 2.03	52.72 ± 0.32
	B7B1	63.07 ± 2.03	58.39 ± 4.21	63.07 ± 2.03	58.99 ± 2.43
	B7B6	64.07 ± 2.03	57.39 ± 4.21	64.07 ± 2.03	56.99 ± 2.43
	B7A1A5	63.84 ± 3.98	56.57 ± 7.05	63.84 ± 3.98	56.63 ± 3.96
	B7A1B1	63.84 ± 1.29	58.99 ± 4.28	63.84 ± 1.29	59.6 ± 3.35
	B7A1B6	64.07 ± 1.24	56.92 ± 3.02	64.07 ± 1.24	56.81 ± 2.34
	B7A5B1	64.07 ± 2.03	57.39 ± 4.21	64.07 ± 2.03	56.99 ± 2.43
	B7A5B6	65.70 ± 2.70	61.15 ± 5.54	65.70 ± 2.70	58.08 ± 2.54
	B7B1B6	61.98 ± 1.01	55.26 ± 1.10	61.98 ± 1.01	56.11 ± 0.80
KNN	A1	69.34 ± 1.58	73.61 ± 4.73	69.34 ± 1.58	60.31 ± 0.49
	A5	59.12 ± 1.43	51.54 ± 2.82	59.12 ± 1.43	53.63 ± 0.21
	B1	66.42 ± 1.36	63.23 ± 2.14	66.42 ± 1.36	62.96 ± 2.59
	B6	67.15 ± 1.86	63.64 ± 2.90	67.15 ± 1.86	61.20 ± 2.41
	**B7**	**71.53 ± 1.80**	**73.65 ± 2.93**	**71.53 ± 1.80**	**72.14 ± 2.43**
	B7A1	62.68 ± 2.39	58.07 ± 3.27	62.68 ± 2.39	58.65 ± 2.53
	B7A5	60.58 ± 2.03	55.56 ± 5.39	60.58 ± 2.03	56.62 ± 4.21
	B7B1	60.58 ± 2.03	55.56 ± 5.39	60.58 ± 2.03	56.62 ± 4.21
	B7A1	60.46 ± 2.94	54.5 ± 4.02	60.46 ± 2.94	55.69 ± 2.9
	B7A1A5	62.67 ± 0.93	58.46 ± 0.73	62.67 ± 0.93	59.02 ± 0.49
	B7A1B1	62.56 ± 2.25	59.58 ± 2.18	62.56 ± 2.25	60.12 ± 1.75
	B7A1B6	62.21 ± 2.32	58.88 ± 2.78	62.21 ± 2.32	59.59 ± 2.57
	B7A5B1	60.46 ± 2.94	54.50 ± 4.02	60.46 ± 2.94	55.69 ± 2.90
	B7A5B6	62.09 ± 3.00	55.70 ± 5.04	62.09 ± 3.0	56.04 ± 2.69
	B7B1B6	63.37 ± 2.94	60.13 ± 3.38	63.37 ± 2.94	60.44 ± 2.52
NB	A1	51.09 ± 1.14	47.74 ± 4.3	51.09 ± 1.14	49.17 ± 1.99
	A5	54.74 ± 2.71	54.25 ± 2.37	54.74 ± 2.71	54.49 ± 2.52
	B1	63.50 ± 1.14	60.87 ± 2.7	63.50 ± 1.14	61.53 ± 3.70
	B6	65.69 ± 0.47	62.95 ± 3.22	65.69 ± 0.47	63.27 ± 0.96
	**B7**	**77.37 ± 3.41**	**79.82 ± 3.48**	**77.37 ± 3.41**	**77.90 ± 3.33**
	B7A1	57.09 ± 3.44	65.37 ± 3.98	57.09 ± 3.44	58.02 ± 3.33
	B7A5	58.96 ± 3.40	64.57 ± 3.1	58.96 ± 3.40	60.05 ± 3.32
	B7B1	58.96 ± 3.40	64.57 ± 3.1	58.96 ± 3.40	60.05 ± 3.32
	B7B6	57.44 ± 2.28	64.74 ± 2.71	57.44 ± 2.28	58.47 ± 2.21
	B7A1A5	58.49 ± 1.71	66.37 ± 2.93	58.49 ± 1.71	59.44 ± 1.55
	B7A1B1	57.91 ± 3.90	65.97 ± 4.45	57.91 ± 3.90	58.86 ± 3.79
	B7A1B6	55.82 ± 5.53	63.34 ± 5.10	55.82 ± 5.53	56.83 ± 5.45
	B7A5B1	57.44 ± 2.28	64.74 ± 2.71	57.44 ± 2.28	58.47 ± 2.21
	B7A5B6	57.56 ± 2.57	62.73 ± 1.72	57.56 ± 2.57	58.67 ± 2.56
	B7B1B6	57.91 ± 4.79	64.56 ± 4.38	57.91 ± 4.79	58.98 ± 4.69

Significance values are in bold.

### Results of single LSTM models

Building a DL model based on the best combination of feature sets is expected to achieve better results. Our data are divided into medically related feature sets including A1, A5, B1, B6, and B7. Different feature sets can contribute differently to the classifier performance, and different combinations of feature sets can affect the role of every individual feature in the resulting fused set. In this experiment, we explore the role of time series data and deep LSTM models to improve the performance of the resulting classifier. In addition, we explore the role of fusing different feature sets. We aim to explore the best combination of features that achieve the best results with the LSTM. Therefore, investigating the performance of the LSTM when dealing with these different combinations of feature sets. As shown in Table 3, we find that our model’s testing accuracy was enhanced to 80.25 based on the B7 modality alone. These results are statistically significantly better than the RF classifier (*P-*value < 0.001). Different fusion of feature sets did not achieve good performance using singe LSTM model. This means that single LSTM model alone is not able to benefit from large number of time series features.

**Table 3:** Performance of LSTM model with six visits timeseries data.

Feature sets	Accuracy	Precision	Recall	F1-score
A1	63.65 ± 3.618	54.86 ± 2.149	63.65 ± 3.62	54.33 ± 1.62
A5	59.1 ± 6.184	59.29 ± 7.736	59.1 ± 6.18	59.4 ± 7.47
B1	64.96 ± 5.211	64.83 ± 3.845	64.96 ± 5.21	64.12 ± 2.96
B6	60.29 ± 5.792	60.70 ± 6.031	60.29 ± 5.79	60.60 ± 6.25
**B7**	**80.25 ± 3.131**	**80.68 ± 2.909**	**80.25 ± 3.13**	**80.42 ± 3.05**
B7A1	66.42 ± 2.636	64.23 ± 1.836	66.42 ± 2.64	63.32 ± 2.47
B7A5	79.56 ± 5.647	80.54 ± 3.888	79.56 ± 5.65	79.85 ± 5.27
B7B1	77.40 ± 2.29	77.16 ± 1.954	77.47 ± 2.29	77.91 ± 2.19
B7B6	76.79 ± 3.988	77.17 ± 3.386	76.79 ± 3.99	76.93 ± 3.66
B7A1A5	74.89 ± 4.829	74.32 ± 5.379	74.89 ± 4.83	74.24 ± 5.38
B7A1B1	66.13 ± 3.498	66.96 ± 0.541	66.13 ± 3.5	66.2 ± 2.87
B7A1B6	69.92 ± 4.722	68.97 ± 3.736	69.92 ± 4.72	69.01 ± 4.49
B7A5B1	73.14 ± 6.205	74.96 ± 3.953	73.14 ± 6.2	73.64 ± 5.86
B7A5B6	71.82 ± 8.905	71.78 ± 6.994	71.82 ± 8.9	71.77 ± 8.55
B7B1B6	73.57 ± 6.02	73.71 ± 5.148	73.57 ± 6.02	73.55 ± 5.78
B7A1A5B1	73.72 ± 4.58	74.27 ± 5.25	73.72 ± 4.58	73.67 ± 4.7
B7A1A5B6	69.34 ± 6.066	68.11 ± 6.481	69.34 ± 6.07	68.38 ± 6.19
B7A1B1B6	70.07 ± 3.907	68.6 ± 3.201	70.07 ± 3.91	68.69 ± 3.59
B7A5B1B6	71.16 ± 6.585	71.96 ± 5.649	71.16 ± 6.58	71.44 ± 6.46
B7A1A5B1B6	72.26 ± 9.424	71.0 ± 7.584	72.26 ± 9.42	70.76 ± 9.38

Significance values are in bold.

**Figure 7:**
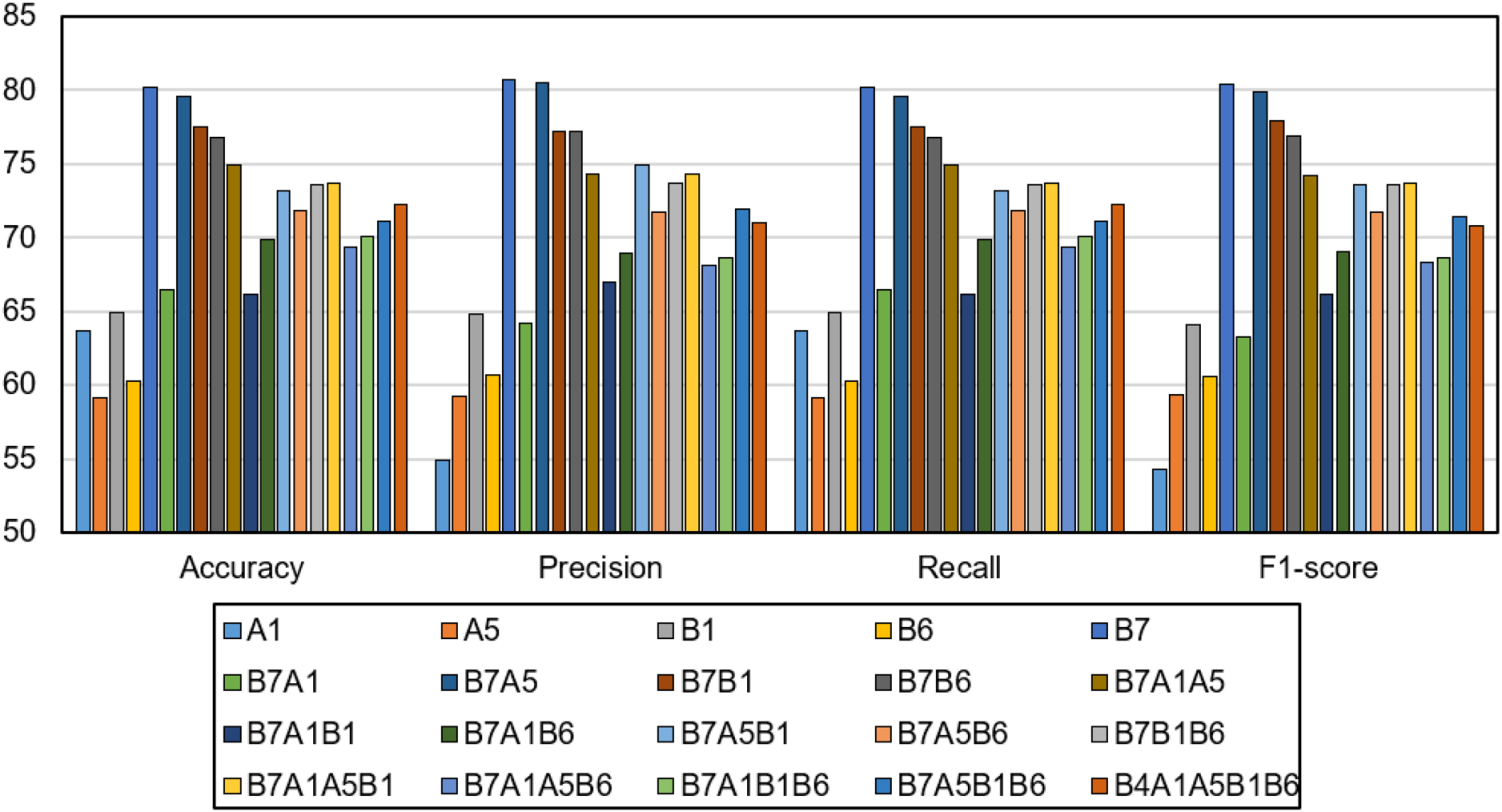
Performance of the best performing feature sets with single LSTM models.

For example, the combination of the five feature sets (i.e., B7/A1/A5/B1/B6) resulted in low performance of 72.26, 71.0, 72.26, and 70.76 for accuracy, precision, recall, and F1-score, respectively. However, the combination of three feature sets (i.e., B7/A1/A5) only resulted in better performance compared to the five feature sets combination (i.e., 74.89, 74.32, 74.89, 74.24 for accuracy, precision, recall, and F1-score, respectively). Another example, the combination of two feature sets only (i.e., B7/A5) resulted in better performance compared to the three feature sets combination (i.e., 79.56, 80.54, 79.56, and 79.85 for accuracy, precision, recall, and F1-score, respectively). In summary, the LSTM model based on B7 along has statistically significantly achieved better results compared to other fusions (*P-*value < 0.001). This means that the LSTM model is simple enough to be able to learn the complex temporal patterns in dataset with larger number of time series features. Figure 7 shows a comparison among different LSTM models. In the next experiment, we build stacking ensembles of simple baseline LSTM models, where each model is based on a single modality. We explore the role of ensembles to improve the performance of the resulting models and to build robust and stable classifiers. Different meta learners are explored.

### Results of stacking deep ensemble models

Ensemble models are expected to improve the performance of base models. In this experiment, we explore the results of ensembles of deep LSTM models. In addition, we explored so many experiments to check the role of fusing different feature sets to enhance the performance of the DL models. Note that fusing of different feature sets creates heterogeneous ensemble models, which theoretically enhances the performance of the resulting models. This hypothesis is proved in this experiment because the results of ensemble models outperform the results of other base LSTM models. Table 4 shows results of different deep LSTM ensemble models based on different combinations of feature sets and using different meta classifiers including SVM, LR, and RF.

**Table 4:** Performance of the stacked deep LSTM ensemble model.

Models	Fused feature sets	Accuracy	Precision	Recall	F1-score
Stacking SVM	A1/A5/B1/B6/B7	69.34 ± 2.83	68.63 ± 3.05	69.34 ± 2.83	68.84 ± 3.01
	**A5/B1/B6/B7**	**78.10 ± 1.82**	**78.18 ± 1.85**	**78.10 ± 1.82**	**76.42 ± 1.84**
	B7/B1/B6	77.37 ± 2.31	77.44 ± 3.03	77.37 ± 2.31	75.5 ± 2.7
	**B7/B1**	**80.37 ± 1.94**	**80.44 ± 2.56**	**80.37 ± 1.94**	**80.5 ± 2.34**
	Best two feature sets	72.81 ± 2.17	71.33 ± 2.01	72.81 ± 2.17	70.92 ± 2.12
	B7A5/B7B1/B7B6	76.46 ± 2.14	76.92 ± 2.22	76.46 ± 2.14	76.54 ± 2.07
	**B7A5/B7B6**	**82.02 ± 1.39**	**82.25 ± 1.7**	**82.02 ± 1.39**	**82.12 ± 1.34**
	B7A5/B7B1	80.44 ± 2.15	80.93 ± 2.33	80.44 ± 2.15	80.61 ± 2.18
	Best three feature sets	75.91 ± 2.12	76.34 ± 2.03	75.91 ± 2.12	76.09 ± 2.06
	B7A1A5/B7A1B6/B7A5B1/B7A5B6/B7B1B6	73.72 ± 1.43	73.72 ± 1.65	73.72 ± 1.43	73.72 ± 1.41
	B7A1A5/B7A5B1/B7A5B6/ B7B1B6	73.72 ± 0.97	75.12 ± 1.56	73.72 ± 0.97	74.17 ± 1.1
	B7A5B1/B7A5B6/B7B1B6	73.72 ± 2.19	74.35 ± 2.04	73.72 ± 2.19	73.97 ± 2.09
	B7A5B1/B7A5B6	72.99 ± 1.53	74.2 ± 1.54	72.99 ± 1.53	73.41 ± 1.54
	B7A1A5/B7B1B6	74.45 ± 1.53	77.5 ± 1.54	74.45 ± 1.53	75.1 ± 1.54
	B7A5B1/B7B1B6	76.64 ± 2.79	77.22 ± 2.58	76.64 ± 2.79	76.87 ± 2.68
	Best four feature sets	75.91 ± 2.67	75.39 ± 2.84	75.91 ± 2.67	75.54 ± 2.7
	B7A1A5B1/B7A1A5B7/B7A1B1B6	73.72 ± 2.43	74.02 ± 2.82	73.72 ± 2.43	73.86 ± 2.65
Stacking LR	A1/A5/B1/B6/B7	70.98 ± 1.43	70.77 ± 1.45	70.98 ± 1.43	70.81 ± 0.94
	**A1/B1/B6/B7**	**78.10 ± 1.35**	**78.18 ± 1.82**	**78.10 ± 1.35**	**76.42 ± 1.68**
	B7/B1B6	75.91 ± 1.81	76.05 ± 2.95	75.91 ± 1.81	75.98 ± 2.29
	B7/B1	79.74 ± 2.79	79.86 ± 3.83	79.74 ± 2.79	79.02 ± 3.62
	Best two feature sets	75.91 ± 2.38	75.26 ± 2.42	75.91 ± 2.38	75.24 ± 2.37
	B7A5/B7B1/B7B6	78.46 ± 2.31	78.46 ± 2.60	78.46 ± 2.31	78.4 ± 2.50
	B7A5/B7B6	79.56 ± 1.63	79.37 ± 2.35	79.56 ± 1.63	79.45 ± 1.85
	B7A5/B7B1	77.37 ± 2.49	78.11 ± 2.59	77.37 ± 2.49	77.64 ± 2.4
	Three feature sets	75.18 ± 2.14	76.15 ± 1.94	75.18 ± 2.14	75.52 ± 2.06
	B7A1A5/B7A1B6/B7A5B1/B7A5B6/B7B1B6	74.45 ± 0.74	74.59 ± 0.9	74.45 ± 0.74	74.52 ± 0.78
	B7A1A5/B7A5B1/B7A5B6/B7B1B6	72.26 ± 0.55	74.13 ± 1.12	72.26 ± 0.55	72.82 ± 0.63
	B7A5B1/B7A5B6/B7B1B6	72.99 ± 1.5	73.82 ± 1.8	72.99 ± 1.5	73.31 ± 1.57
	B7A5B1/B7A5B6	72.26 ± 2.38	74.6 ± 2.68	72.26 ± 2.38	72.88 ± 2.58
	B7A1A5/B7B1B6	74.45 ± 2.38	75.25 ± 2.68	74.45 ± 2.38	74.75 ± 2.58
	B7A5B1/B7B1B6	77.37 ± 2.88	78.88 ± 3.3	77.37 ± 2.88	77.79 ± 3.01
	Best four feature sets	75.91 ± 3.72	75.16 ± 3.73	75.91 ± 3.72	75.17 ± 3.72
	B7A1A5B1/B7A1A5B7/B7A1B1B6	75.91 ± 2.83	75.39 ± 3.24	75.91 ± 2.83	75.54 ± 3.12
Stacking RF	A1/A5/B1/B6/B7	72.08 ± 1.94	71.72 ± 1.94	72.08 ± 1.94	71.85 ± 1.84
	A1/B1/B6/B7	75.18 ± 2.15	74.38 ± 2.16	75.18 ± 2.15	74.16 ± 1.99
	B7/B1B6	72.81 ± 3.68	72.05 ± 2.71	72.81 ± 3.68	72.27 ± 3.23
	B7/B1	78.83 ± 2.14	78.91 ± 1.97	78.83 ± 2.14	77.34 ± 2.16
	Best two feature sets	74.82 ± 2.03	73.98 ± 2.18	74.82 ± 2.03	73.92 ± 2.13
	B7A5/B7B1/B7B6	77.19 ± 3.03	77.76 ± 3.46	77.19 ± 3.03	77.33 ± 3.07
	B7A5/B7B6	78.10 ± 1.22	78.79 ± 1.57	78.10 ± 1.22	78.35 ± 1.47
	B7A5/B7B1	76.28 ± 3.11	76.12 ± 3.07	76.28 ± 3.11	76.18 ± 3.06
	Best three feature sets	77.37 ± 2.64	79.58 ± 3.14	77.37 ± 2.64	77.88 ± 3.02
	B7A1A5/B7A1B6/B7A5B1/B7A5B6/B7B1B6	78.46 ± 0.74	78.66 ± 0.9	78.46 ± 0.74	78.55 ± 0.78
	B7A1A5/B7A5B1/ B7A5B6/B7B1B6	76.09 ± 1.93	78.27 ± 2.23	76.09 ± 1.93	76.62 ± 2.18
	B7A5B1/B7A5B6/B7B1B6	71.9 ± 1.70	72.85 ± 1.82	71.9 ± 1.70	72.24 ± 1.73
	B7A5B1/B7A5B6	71.53 ± 2.58	73.31 ± 2.58	71.53 ± 2.58	72.08 ± 2.58
	B7A1A5/B7B1B6	72.44 ± 3.47	73.55 ± 4.3	72.44 ± 3.47	72.82 ± 3.83
	**B7A5B1/B7B1B6**	**79.74 ± 2.14**	**80.48 ± 2.46**	**79.74 ± 2.14**	**79.99 ± 2.41**
	Best four feature sets	75.18 ± 2.83	74.34 ± 2.99	75.18 ± 2.83	74.32 ± 2.95
	B7A1A5B1/B7A1A5B7/B7A1B1B6	73.9 ± 3.38	73.05 ± 3.71	73.9 ± 3.38	73.05 ± 3.57

Significance values are in bold.

In our performance evaluation of the stacking deep ensemble model, we experiment with feature sets based on their individual performance on the single LSTM models. Moreover, we examine the best combination of feature sets that achieved the best results from Tables 2 and 3. According to the result shown in Table 4, we observed that the stacked LSTM models based on SVM meta classifier generally achieve better results than the LR and RF based ensemble models, but these results are not statistically significant. The stacking model with two LSTM baseline classifiers achieves the best testing results, where a separate LSTM model is used with B7A5 and B7B6 fused feature sets. In this experiment, we integrate the early fusion of B7 modality with other feature sets like B6 and A5 with the decision fusion of the two LSTM models. This model achieves testing results of 82.02, 82.25, 82.02, and 82.12 for accuracy, precision, recall, and F1-score, respectively. These results are statistically significantly better than classical ML models and single LSTM models (*P*-value < 0.001). We noticed that using a single feature set with the base LSTM models achieved a lower result.

**Figure 8:**
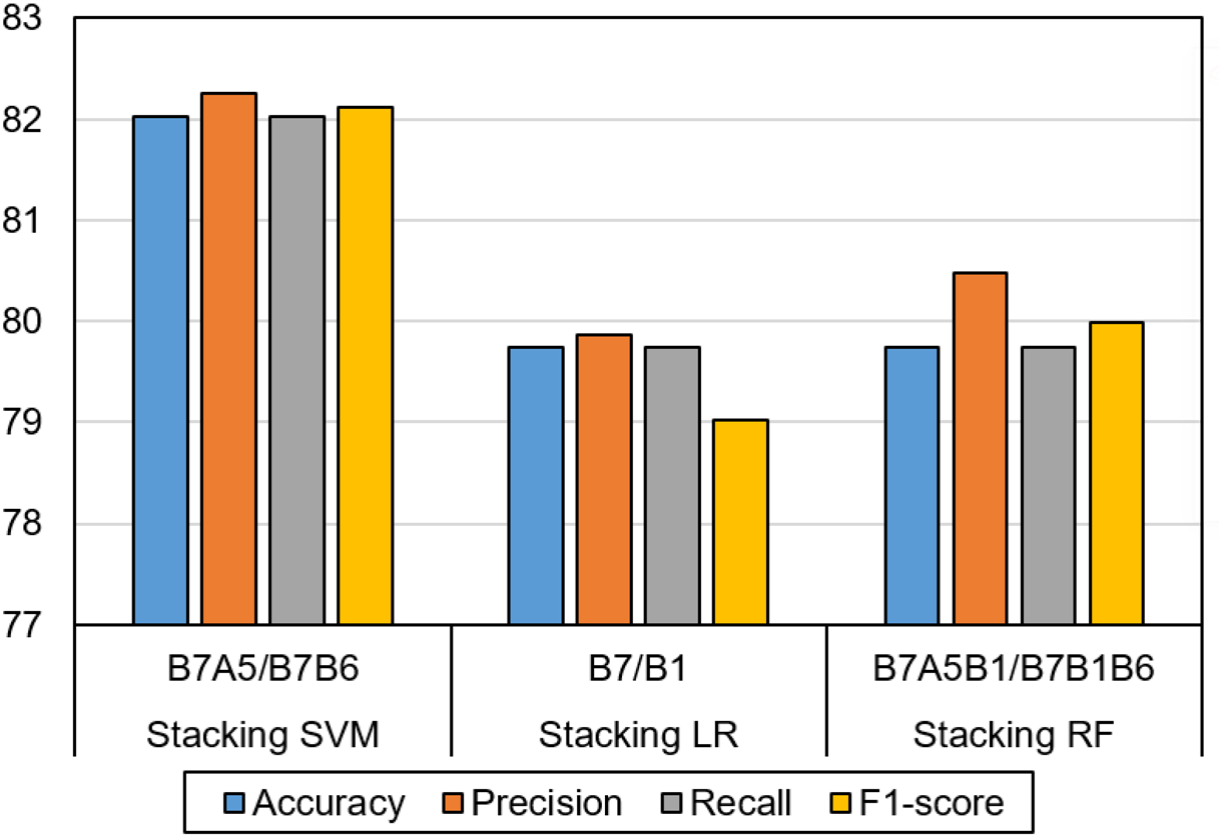
The best stacking ensemble models with different meta-learners.

For example, building a stacking ensemble of two base line classifiers, where each classifier is based on a single modality (i.e., B7 and B1), resulted in lower results compared to the previous experiment (i.e., 80.37, 80.44, 80.37, and 80.5 for accuracy, precision, recall, and F1-score, respectively). On the other hand, the combination of several baseline LSTM models which are based on the early fusion of multiple feature sets resulted in worse results. For example, in an experiment, we combined five LSTM models where each model is based on an early fusion of three feature sets (i.e., B7A1A5, B7A1B6, B7A5B1, B7A5B6, B7B1B6), and this ensemble achieved bad results of 73.72, 73.72, 73.72, and 73.72 for accuracy, precision, recall, and F1-score, respectively. As a result, even ensemble of multiple DL models could boost the performance, but wise selection of the number of base classifiers, the early fusion of feature sets, and the selection of meta learners is crucial. This is an art, where there is no theory or heuristics that could govern this behavior and predetermine the best settings for better ensemble architecture. The same pattern in results has been noticed for stacking ensemble with LR and stacking ensemble with RF. For the Stacking with LR, the best performing model (i.e., 79.74, 79.86, 79.74, and 79.02 for accuracy, precision, recall, and F1-score, respectively) was based on two LSTM base models each was based on a single modality (i.e., B7 and B1). Increasing the number of feature sets in the early fusion and increasing the number of base LSTM classifiers did not achieve better results. Stacking with RF achieved the best results with two baseline LSTM classifiers each one was based on an early fusion of three feature sets (i.e., B7A5B1 and B7B1B6), and the performance was 79.74, 80.48, 79.74, and 79.99 for accuracy, precision, recall, and F1-score, respectively. Figure 8 shows a comparison of the three ensemble models.

Thus, according to all our experimentations, we noticed an increasing performance in terms of accuracy metrics when relying on stacked LSTM ensemble models over regular ML and LSTM models. According to the testing performance results, the accuracy of the best models has increased from 77.83 in regular SVM to 80.25 in the LSTM, and finally 82.02 in the stacking ensemble models. These results proved that the stacking ensemble models outperformed all other ML and simple LSTM classification models. Figure 8 clarifies the comparison between the best model of stacking ensemble models with different meta learners.

### Comparison with the literature

In this section, we compare the proposed model with the state-of-the-art literature of ensemble models for AD early detection as shown on Table 5. In Ref.^79^, authors proposed a transfer learning model based on ResNet18 architecture to detect the LMCT patient. They used PET and MRI images. In Ref.^80^, the study explored the role of ensemble model with majority voting to build a set of binary classifiers to solve the problems of CN vs. AD, CN vs. MCI, and AD vs. MCI based on MRI modality. In Ref.^81^, an ADNI dataset from MRI and PET modalities has been used to train the MobileNet to detect AD patients based on the early fusion of features from the two modalities. In Ref.^82^, the study a large ADNI dataset of MRI images to optimize the XGB classifier to detect AD, and in Ref.^83^, another ADNI dataset has been used to train the VGG-16 deep learning model to detect AD. Muhammed Niyas and Thiyagarajan^72^ proposed a dynamic ensemble classifier for AD detection. The was based on two different classifiers of RF and bagging of decision trees. The study early fused the MRI, PET, CSF, CS, and demographics (i.e., age, sex, education), and achieved CV balanced accuracy of 87% and testing balanced accuracy of 82% based on an ADNI dataset of CN (523), MCI (872), AD (342). Syed et al.^73^ proposed an MCI detection ensemble classifier based on the weighted voting of the two base classifiers of LR and SVM. The task was implemented as CN vs. MCI binary classification based on a Fig share dataset of CN (242), MCI (91). The study investigated the role of CSF protein biomarkers to detect AD and achieved testing accuracy of 95.5%. Pan et al.^74^ proposed a two-levels ensemble model for detecting AD. The first live had seven SVM classifiers and the second level has three SVM classifiers. The study has utilized an FDG-PET dataset from ADNI database of CN (90), sMCI (44), pMCI (44), and AD (94). To achieve diversity among the base classifiers, seven LASSO feature selection models have been used, one with each base classifier to select a different feature set. for a CN vs. AD task, the model achieved an accuracy of 91.9%, for the CN vs. MCI task, the model achieved an accuracy of 83.2%, and for the sMCI vs. pMCI the model achieved 72.3%. Ahmed et al.^75^ proposed a deep stacking ensemble model of three CNN models with different architectures and SoftMax meta learner to detect AD. The study was based on the sMRI data collected from ADNI and Gwangju Alzheimer's and Related Dementia, Gwangju, South Korea (GARD). Using ADNI data, the model achieved testing accuracy, precision, recall, and F1-score of 85.6, 85.5, and 85.5, respectively. With the GARD data, the model achieved a testing performance of 90.1, 89.9, and 90 for accuracy, precision, and recall, respectively.

**Table 5:** Comparison with literature studies.

References	Data set	Subjects	Feature set	Base classifiers	Diversity source	Fusion method	Ensemble technique	CV	Target	Performance
Ours	NACC	CN (229) and AD (456)	B7A5/B7B6	LSTM	Diverse LSTM on 4 different feature sets	Late feature fusion	Stacking (SVM)	Split 80:20 for train: test	AD,CN	82.02/82.25/82.02/82.12
^79^ 2023	ADNI	EMCI (2150) LMCI (1870)	PET and MRI	ResNet18 (3-in-Channel)	–	Early feature fusion	–	Split 70:30 for train:test	EMCI and LMCI	73.90/66.74
^80^ 2023	ADNI	CN (44), MCI (84) and AD (22)	MRI	transfer learning-based structural	Diverse transfer learning based structural	Late feature fusion	Ensemble of majority voting	Split 70:30 for train:test	CN vs AD	96
									CN vs MCI	72
									AD vs MCI	70
^81^ 2023	ADNI	CN (321) and AD (136)	PET,MRI	MobileNet	–	Early feature fusion	–	Split 80:20 for train:test	AD vs CN	81.94/78.95
^82^ 2022	ADNI	MildDemented (896) ModerateDemented (64) NonDemented (200) VeryMildDemented (2240)	MRI	XG Boost classifier	Different tree architectures	Early feature fusion	Boosting	Split 80:20 for train:test	Binary	73
									Multiclass	76
^83^ 2022	ADNI	MildDemented (896) ModerateDemented (64) NonDemented (200) VeryMildDemented (2240)	MRI	VGG-16	–	Early feature fusion	–	Split 80:20 for train:test	Multiclass	75
^72^ (2021)	ADNI	CN (523), MCI (872), AD (342)	MRI, PET, CSF, CS, (age, sex, education)	2 classifiers [RF + BDT]	Diverse ML models	5 diverse data types (Early fusion)	META-DES (DES)	Split 80:20 for train:test + stratified 10-CV on train	CN versus MCI versus AD (balanced accuracy)	82/–/80/–/–
^73^ (2020)	Figshare	CN (242), MCI (91)	CSF protein biomarkers	2 classifiers [LR + linear SVM]	Diverse ML models	–	Weighted average (SES)	Stratified split 80:20 for train: test + 5-CV on train	CN vs. MCI	95.5/–/95.7/–/97.9
^74^ (2019)	ADNI	CN (90), sMCI (44), pMCI (44), AD (94)	ADNI’s Post processed FDG-PET	*Level 1*: 7 classifiers [SVM] + *Level 2*: 3 classifiers [SVM]	7 LASSO FS on 7 different feature sets	Region based and connectivity between regions-based features (late fusion)	Maximum mean square error (mMsE) of 7 SVMs + majority voting of 3 SVMs	10 times repeated 10-CV	CN vs. AD	–/–/–/–/–
									CN vs. MCI	–/–/–/–/–
									sMCI vs. pMCI	–/–/–/–/–
^75^ (2019)	ADNI, GARD	ADNI: CN (129), AD (77), GARD: AD (81), CN (171)	sMRI	3 classifiers [CNNs] with different architectures	Three feature sets from TVPLH, TVPRH, TVPLHRH	Subsets of features (early fusion)	Stacking with SoftMax meta classifier	80:20 for train:test	CN vs. AD on ADNI	85.6/85.5/85.5/85.5/–
									CN vs. AD on GARD	90.1/89.9/90.0/90.0/–
^76^ (2018)	ADNI	*Training*: CN (60), sMCI (60), pMCI (60), AD (60), *Testing*: CN (40), sMCI (40), pMCI (40), AD (40)	MRI, MMSE, age, CSF	5 classifiers [RF]	Diverse input data (DID1)	Subsets of features (early and late fusion)	Majority voting (SES)	Repeated 10-CV	CN vs. sMCI vs. pMCI vs. AD	61.9/60.2/61.9/–/60.5
^77^ (2018)	ADNI	CN (60), sMCI (60), pMCI (60), AD (60)	MRI, age, gender, MMSE	4 classifiers [SVM]	Diverse input data (DID2)	Subsets of features (early and late fusion)	Static classifier selection (SES)	Split 80:20 for train:test + stratified 4-CV on train	CN vs. sMCI vs. pMCI vs. AD	52.9/–/–/–/79.6
^78^ (2018)	ADNI	CN (60), sMCI (60), pMCI (60), AD (60)	Preprocessed MRI, age, gender, MMSE	150 classifiers [decision tree]	Different tree architectures	–	Boosting decision tree ensemble	10-CV	CN vs. sMCI vs. pMCI vs. AD	56.3/–/–/–/–

In Ref.^76^, the study proposed an AD progression detection model. The model has been implemented as a majority voting ensemble of five RF classifiers. The study tested the early and late fusion of MRI, MMSE, age, CSF features. Different ADNI datasets have been used for training and testing, i.e., training: CN (60), sMCI (60), pMCI (60), AD (60), and testing: CN (40), sMCI (40), pMCI (40), AD (40). For the multiclass classification task of CN vs. sMCI vs. pMCI vs. AD, the model achieved a performance of 61.9% for accuracy, 60.2 for precision, and 61.9 for recall. In comparison with the literature, our study proposed many unique features both in machine learning and in medical domains. In the medical domain, the proposed study is based on multivariate time series data to predict AD progression. The study built a stacking ensemble model of multiple LSTM deep learning models. Each modality and combination of feature sets have been tested to select the best fusion of feature sets that achieved the best results. Our study is based on cheap features to predict the AD disease which make our model applicable in real environments where MRI scans are not available. Our proposed model achieved promising results compared to the literature, even though it has used the least number of cost-effective feature sets^66^. We utilized Bayesian optimizer and grid search to optimize the LSTM base classifiers and the SVM meta learner, respectively. Our results have been done on the NACC dataset, where no such studies have been done before.

## Limitations and future directions

Our study implemented and tested an advanced deep LSTM based stacking ensemble model for AD detection. The study advanced the literature of ensemble modeling and used the NACC multivariate time series data. However, the study has some limitations that should be covered in future works. First, we will extend the current study by adding explainability features which improves the model understandability and increases the trust of domain experts^86^. The explainability can be improved by reducing the number of input features, we will explore different feature selection techniques on each feature set^87^. Second, the study has been totally trained and tested based on the NACC dataset. We did not test the proposed model on data collected from other sources like ADNI. This is called external validation which measures the model’s reproducibility feature. In future studies, we will explore the performance of the proposed model on an external dataset. Finally, we will explore the effect of adding neuroimaging modalities (MRI, PET, CT etc. images) as input to the base classifiers.

## Conclusion

In this paper, we proposed a novel stacking deep ensemble classifier based on the deep LSTM base classifier and LR meta model. The study was based on multivariate time series data to predict AD. To better learn these time series data LSTM deep learning models have been used. A separate LSTM model has been optimized using Bayesian optimizer to select the best hyperparameters for a specific modality. Heterogeneous feature sets have been used with different LSTM base models to build the stacking ensemble model. We discovered that LSTM base models outperformed other classical machine learning models. In addition, the combined heterogeneous LSTM models based on different feature sets to build the stacking ensemble have improved the performance of each base LSTM classifier. The NACC dataset has been used to explore the performance of these models. The data has been divided into training and testing from the first beginning before data preprocessing steps; this decision prevented the data leakage problem which causes ML models to achieve over optimistic testing results. The training data has been used to train, validate, and optimize the models using cross-validation technique. Although the resulting ensemble achieved the best and most stable results, these models are black boxes where physicians do not understand why the model has taken specific decisions. In future studies, we will extend the proposed model to provide explainability for its local and global decisions.

### Supplementary Information

Supplementary Table S1.

## Data Availability

The datasets generated and/or analyzed during the current study are available in the University of Washington's National Alzheimer’s Coordinating Center (NACC) repository, https://naccdata.org/nacc-collaborations/about-nacc.

## References

[1] Hao X (2020). Multi-modal neuroimaging feature selection with consistent metric constraint for diagnosis of Alzheimer’s disease. Med. Image Anal..

[2] Alzheimer's Association (2017). 2017 Alzheimer’s disease facts and figures. Alzheimer’s Dement..

[3] Mirzaei G, Adeli H (2022). Machine learning techniques for diagnosis of alzheimer disease, mild cognitive disorder, and other types of dementia. Biomed. Signal Process. Control.

[4] Nogay HS, Adeli H (2020). Machine learning (ML) for the diagnosis of autism spectrum disorder (ASD) using brain imaging. Rev. Neurosci..

[5] Vuttipittayamongkol P, Elyan E (2020). Improved overlap-based undersampling for imbalanced dataset classification with application to epilepsy and parkinson’s disease. Int. J. Neural Syst..

[6] Amezquita-Sanchez JP, Mammone N, Morabito FC, Adeli H (2021). A new dispersion entropy and fuzzy logic system methodology for automated classification of dementia stages using electroencephalograms. Clin. Neurol. Neurosurg..

[7] Acharya UR, Oh SL, Hagiwara Y, Tan JH, Adeli H, Subha DP (2018). Automated EEG-based screening of depression using deep convolutional neural network. Comput. Methods Progr. Biomed..

[8] Heo J, Yoon JG, Park H, Kim YD, Nam HS, Heo JH (2019). Machine learning–based model for prediction of outcomes in acute stroke. Stroke.

[9] Martí-Juan G, Sanroma-Guell G, Piella G (2020). A survey on machine and statistical learning for longitudinal analysis of neuroimaging data in Alzheimer’s disease. Comput. Methods Progr. Biomed..

[10] Chételat G (2018). Multimodal neuroimaging in Alzheimer’s disease: Early diagnosis, physiopathological mechanisms, and impact of lifestyle. J. Alzheimer’s Dis..

[11] Gómez-Sancho M, Tohka J, Gómez-Verdejo V (2018). Comparison of feature representations in MRI-based MCI-to-AD conversion prediction. Magn. Reson. Imaging.

[12] Yamanakkanavar N, Choi JY, Lee B (2020). MRI segmentation and classification of human brain using deep learning for diagnosis of Alzheimer ’ s disease : A survey. Sensors.

[13] Li H, Habes M, Wolk DA, Fan Y (2019). A deep learning model for early prediction of Alzheimer’s disease dementia based on hippocampal MRI. Alzheimer’s Dement..

[14] Ben Rabeh, A., Benzarti, F., & Amiri, H. Diagnosis of Alzheimer diseases in early step using SVM (support vector machine), in *2016 13th International Conference on Computer Graphics, Imaging and Visualization (CGiV)*, 364–367 (2016).

[15] Ferreira LK (2017). Support vector machine-based classification of neuroimages in Alzheimer’s disease: direct comparison of FDG-PET, rCBF-SPECT and MRI data acquired from the same individuals. Braz. J. Psychiatry.

[16] Moore PJ, Lyons TJ, Gallacher J (2019). Random forest prediction of Alzheimer’s disease using pairwise selection from time series data. PLoS ONE.

[17] Wang H (2019). Ensemble of 3D densely connected convolutional network for diagnosis of mild cognitive impairment and Alzheimer’s disease. Neurocomputing.

[18] Pan D, Zeng A, Jia L, Huang Y, Frizzell T, Song X (2020). Early detection of alzheimer’s disease using magnetic resonance imaging: A novel approach combining convolutional neural networks and ensemble learning. Front. Neurosci..

[19] Shi J, Zheng X, Li Y, Zhang Q, Ying S (2018). Multimodal neuroimaging feature learning with multimodal stacked deep polynomial networks for diagnosis of Alzheimer’s disease. IEEE J. Biomed. Heal. Inf..

[20] Farooq, A., Anwar, S., Awais, M. & Rehman, S. A deep CNN based multi-class classification of Alzheimer’s disease using MRI, in *2017 IEEE International Conference on Imaging systems and techniques (IST)*, 1–6 (2017).

[21] Jain R, Jain N, Aggarwal A, Hemanth DJ (2019). Convolutional neural network based Alzheimer’s disease classification from magnetic resonance brain images. Cogn. Syst. Res..

[22] Nguyen M, He T, An L, Alexander DC, Feng J, Yeo BTT (2020). Predicting Alzheimer’s disease progression using deep recurrent neural networks. Neuroimage.

[23] Lee G (2019). Predicting Alzheimer’s disease progression using multi-modal deep learning approach. Sci. Rep..

[24] Abuhmed T, El-Sappagh S, Alonso JM (2021). Robust hybrid deep learning models for Alzheimer’s progression detection. Knowl. Based Syst..

[25] Arafa DA, Moustafa HE-D, Ali-Eldin AMT, Ali HA (2022). Early detection of Alzheimer’s disease based on the state-of-the-art deep learning approach: A comprehensive survey. Multimed. Tools Appl..

[26] El Sappagh S, Alonso JM, Islam SMR, Sultan AM (2021). A multilayer multimodal detection and prediction model based on explainable artificial intelligence for Alzheimer’s disease. Sci. Rep..

[27] Polikar R (2008). An ensemble based data fusion approach for early diagnosis of Alzheimer’s disease. Inf. Fusion.

[28] El-Rashidy N, El-Sappagh S, Abuhmed T, Abdelrazek S, El-Bakry HM (2020). Intensive care unit mortality prediction: An improved patient-specific stacking ensemble model. IEEE Access.

[29] Zounemat-Kermani M, Batelaan O, Fadaee M, Hinkelmann R (2021). Ensemble machine learning paradigms in hydrology: A review. J. Hydrol..

[30] Alickovic, E., Subasi, A., & Initiative, A. D. N. Automatic detection of alzheimer disease based on histogram and random forest, in *International Conference on Medical and Biological Engineering*, 91–96 (2019).

[31] Ortiz A, Munilla J, Gorriz JM, Ramirez J (2016). Ensembles of deep learning architectures for the early diagnosis of the Alzheimer’s disease. Int. J. Neural Syst..

[32] An N, Ding H, Yang J, Au R, Ang TFA (2020). Deep ensemble learning for Alzheimer’s disease classification. J. Biomed. Inform..

[33] El-Sappagh S, Abuhmed T, Islam SMR, Kwak KS (2020). Multimodal multitask deep learning model for Alzheimer’s disease progression detection based on time series data. Neurocomputing.

[34] El-Sappagh S (2021). Alzheimer’s disease progression detection model based on an early fusion of cost-effective multimodal data. Futur. Gener. Comput. Syst..

[35] Ramírez J (2018). Ensemble of random forests one vs. rest classifiers for MCI and AD prediction using ANOVA cortical and subcortical feature selection and partial least squares. J. Neurosci. Methods.

[36] El-Sappagh S, Saleh H, Ali F, Amer E, Abuhmed T (2022). Two-stage deep learning model for Alzheimer’s disease detection and prediction of the mild cognitive impairment time. Neural Comput. Appl..

[37] El-Sappagh S, Abuhmed T, Riazul Islam SM, Kwak KS (2020). Multimodal multitask deep learning model for Alzheimer’s disease progression detection based on time series data. Neurocomputing.

[38] Fathi S, Ahmadi M, Dehnad A (2022). Early diagnosis of Alzheimer’s disease based on deep learning: A systematic review. Co mput. Biol. Med..

[39] Woźniak M, Graña M, Corchado E (2014). A survey of multiple classifier systems as hybrid systems. Inf. Fusion.

[40] Yao D, Calhoun VD, Fu Z, Du Y, Sui J (2018). An ensemble learning system for a 4-way classification of Alzheimer’s disease and mild cognitive impairment. J. Neurosci. Methods.

[41] Farhan S, Fahiem MA, Tauseef H (2014). An ensemble-of-classifiers based approach for early diagnosis of alzheimer’s disease: Classification using structural features of brain images. Comput. Math. Methods Med..

[42] El-Sappagh S, Elmogy M, Ali F, Abuhmed T, Islam SMR, Kwak K-S (2019). A comprehensive medical decision–support framework based on a heterogeneous ensemble classifier for diabetes prediction. Electronics.

[43] Sørensen L, Nielsen M (2018). Ensemble support vector machine classification of dementia using structural MRI and mini-mental state examination. J. Neurosci. Methods.

[44] Loddo A, Buttau S, Di Ruberto C (2022). Deep learning based pipelines for Alzheimer’s disease diagnosis: A comparative study and a novel deep-ensemble method. Comput. Biol. Med..

[45] Ji, H., Liu, Z., Yan, W. Q. & Klette, R. Early diagnosis of Alzheimer’s disease using deep learning, in *Proceedings of the 2nd International Conference on Control and Computer Vision*, 87–91 (2019).

[46] Jabason, E., Ahmad, M. O., & Swamy, M. N. S. Classification of Alzheimer’s disease from MRI data using an ensemble of hybrid deep convolutional neural networks, in *2019 IEEE 62nd International Midwest Symposium on Circuits and Systems (MWSCAS)*, 481–484 (2019).

[47] Kang W, Lin L, Zhang B, Shen X, Wu S, Initiative ADN (2021). Multi-model and multi-slice ensemble learning architecture based on 2D convolutional neural networks for Alzheimer’s disease diagnosis. Comput. Biol. Med..

[48] Zhang P, Lin S, Qiao J, Tu Y (2021). Diagnosis of Alzheimer’s Disease with ensemble learning classifier and 3D convolutional neural network. Sensors.

[49] Ebadi A (2017). Ensemble classification of Alzheimer’s disease and mild cognitive impairment based on complex graph measures from diffusion tensor images. Front. Neurosci..

[50] Choi JY, Lee B (2020). Combining of multiple deep networks via ensemble generalization loss, based on MRI Images, for Alzheimer’s disease classification. IEEE Signal Process. Lett..

[51] Wolpert DH (1992). Stacked generalization. Neural Netw..

[52] Breiman L (1996). Stacked regressions. Mach. Learn..

[53] Kazmaier J, van Vuuren JH (2022). The power of ensemble learning in sentiment analysis. Expert Syst. Appl..

[54] Kaur P, Singh A, Chana I (2022). BSense: A parallel Bayesian hyperparameter optimized Stacked ensemble model for breast cancer survival prediction. J. Comput. Sci..

[55] Abdollahi J, Nouri-Moghaddam B (2022). Hybrid stacked ensemble combined with genetic algorithms for diabetes prediction. Iran. J. Comput. Sci..

[56] Li Z (2022). Developing stacking ensemble models for multivariate contamination detection in water distribution systems. Sci. Total Environ..

[57] Obasi T, Shafiq MO (2022). CARD-B: A stacked ensemble learning technique for classification of encrypted network traffic. Comput. Commun..

[58] Fang X, Liu Z, Xu M (2020). Ensemble of deep convolutional neural networks based multi-modality images for Alzheimer’s disease diagnosis. IET Image Process..

[59] Beekly DL (2007). The national Alzheimer’s coordinating center (NACC) database: the uniform data set. Alzheimer Dis. Assoc. Disord..

[60] Aqeel A (2022). A long short-term memory biomarker-based prediction framework for Alzheimer’s Disease. Sensors.

[61] Jung W, Jun E, Il Suk H (2021). and Alzheimer’s Disease Neuroimaging Initiative, “Deep recurrent model for individualized prediction of Alzheimer’s disease progression”. Neuroimage.

[62] Mehdipour Ghazi M (2019). Training recurrent neural networks robust to incomplete data: Application to Alzheimer’s disease progression modeling. Med. Image Anal..

[63] Lei B (2022). Predicting clinical scores for Alzheimer’s disease based on joint and deep learning. Expert Syst. Appl..

[64] Cui R, Liu M, Initiative N (2019). RNN-based longitudinal analysis for diagnosis of Alzheimer’s Disease. Comput. Med. Imaging Graph..

[65] Morris JC (2006). The uniform data set (UDS): Clinical and cognitive variables and descriptive data from Alzheimer Disease Centers. Alzheimer Dis. Assoc. Disord..

[66] Wang T, Qiu RG, Yu M (2018). Predictive modeling of the progression of Alzheimer’s disease with recurrent neural networks. Sci. Rep..

[67] Donnelly-Kehoe PA, Pascariello GO, Gómez JC (2018). Looking for Alzheimer’s Disease morphometric signatures using machine learning techniques. J. Neurosci. Methods.

[68] Hochreiter S, Schmidhuber J (1997). Long short-term memory. Neural Comput..

[69] Khairalla MA, Ning X, Al-Jallad NT, El-Faroug MO (2018). Short-term forecasting for energy consumption through stacking heterogeneous ensemble learning model. Energies.

[70] Huang L, Jin Y, Gao Y, Thung KH, Shen D (2016). Longitudinal clinical score prediction in Alzheimer’s disease with soft-split sparse regression based random forest. Neurobiol. Aging.

[71] Williams MM, Storandt M, Roe CM, Morris JC (2013). Progression of Alzheimer’s disease as measured by clinical dementia rating sum of boxes scores. Alzheimer’s Dement..

[72] Muhammed-Niyas KP, Thiyagarajan P (2021). Alzheimer’s classification using dynamic ensemble of classifiers selection algorithms: A performance analysis. Biomed. Signal Process. Control.

[73] Syed AH, Khan T, Hassan A, Alromema NA, Binsawad M, Alsayed AO (2020). An ensemble-learning based application to predict the earlier stages of Alzheimer’s disease (AD). IEEE Access.

[74] Pan X, Adel M, Fossati C, Gaidon T, Guedj E (2019). Multilevel feature representation of FDG-PET brain images for diagnosing Alzheimer’s disease. IEEE J. Biomed. Heal. Informatics.

[75] Ahmed S (2019). Ensembles of patch-based classifiers for diagnosis of Alzheimer diseases. IEEE Access.

[76] Dimitriadis SI, Liparas D (2018). Random forest feature selection, fusion and ensemble strategy: Combining multiple morphological MRI measures to discriminate among healhy elderly, MCI, cMCI and alzheimer’s disease patients: From the alzheimer’s disease neuroimaging initiative (ADNI) data. J. Neurosci. Methods.

[77] Nanni L, Lumini A, Zaffonato N (2018). Ensemble based on static classifier selection for automated diagnosis of mild cognitive impairment. J. Neurosci. Methods.

[78] Jin M, Deng W (2018). Predication of different stages of Alzheimer’s disease using neighborhood component analysis and ensemble decision tree. J. Neurosci. Methods.

[79] Odusami M, Maskeliūnas R, Damaševičius R, Misra S (2023). Explainable deep-learning-based diagnosis of Alzheimer’s disease using multimodal input fusion of PET and MRI Images. J. Med. Biol. Eng..

[80] Rallabandi VS, Seetharaman K (2023). Alzheimer's Disease Neuroimaging Initiative (ADNI Classification of cognitively normal controls, mild cognitive impairment and Alzheimer’s disease using transfer learning approach. Biomed. Signal Process. Control.

[81] Ghosh T, Palash MI, Yousuf MA, Hamid MA, Monowar MM, Alassafi MO (2023). A robust distributed deep learning approach to detect Alzheimer’s Disease from MRI images. Mathematics..

[82] Harish, M. V., Dinesh, C., Sasikala, S., Kumar, A. Alzheimer's Disease prediction using machine learning methodologies. In *2022 International Conference on Computer Communication and Informatics (ICCCI)* 1–6. IEEE (2022)

[83] Ganesh, C. H., Nithin, G. S., Akshay, S., Rao, T. V. Multi class Alzheimer disease detection using deep learning techniques. in 2022 *International Conference on Decision Aid Sciences and Applications (DASA)* 470–474. IEEE (2022).

[84] Junaid M, Ali S, Eid F, El-Sappagh S, Abuhmed T (2023). Explainable machine learning models based on multimodal time-series data for the early detection of Parkinson’s disease. Comput. Methods Progr. Biomed..

[85] Rahim N, El-Sappagh S, Ali S, Muhammad K, Del Ser J, Abuhmed T (2023). Prediction of Alzheimer's progression based on multimodal deep-learning-based fusion and visual explainability of time-series data. Inf. Fusion.

[86] El-Sappagh, S., Alonso-Moral, J. M., Abuhmed, T., Ali, F. & Bugarín-Diz, A. Trustworthy artificial intelligence in Alzheimer’s disease: State of the art, opportunities, and challenges. *Artif. Intell. Rev.* 1–148 (2023).

[87] Ali S, Abuhmed T, El-Sappagh S, Muhammad K, Alonso-Moral JM, Confalonieri R, Guidotti R, Del Ser J, Díaz-Rodríguez N, Herrera F (2023). Explainable artificial intelligence (XAI): What we know and what is left to attain trustworthy artificial intelligence. Inf. Fusion.

