# Supplementary material for: Computer aided progression detection model based on optimized deep LSTM ensemble model and the fusion of multivariate time series data
Source: Sci Rep. 2023 Sep 28;13:16336. doi: 10.1038/s41598-023-42796-6 (PMC10539296; doi:10.1038/s41598-023-42796-6)
Supplement: Supplementary file 1 — Supplementary Table S1. [file 41598_2023_42796_MOESM1_ESM.docx]

**Supplementary materials**

**Computer Aided Detection Model Based on Optimized Deep LSTM Ensemble Model and the Fusion of Multimodal Time Series Data**

Hager Saleh^1^, Eslam Amer^2^, Tamer ABUHMED^3^, Amjad Ali^3^, Ala Al-Fuqaha^4*^, Shaker El-Sappagh*^3,5,6^

**Table S1:** Features description

| NACC Categories | Variable Name | Variable Type | Descriptions |
| --- | --- | --- | --- |
| AD stage | CDRGLOB | Ordinal | Global CDR score, which is used to define the AD progression stages |
| Time interval | TI | Continuous | Derived by authors; Time interval between two consecutive visits |
| A1 Subject Demographics | AGE | Continuous | Derived by authors |
|  | SEX | Nominal | Subject’s sex |
|  | RACE | Nominal | Subject’s race |
|  | PRIMLANG | Nominal | Primary language |
|  | EDUC | Ordinal | Years of education |
|  | NACCLIVS | Nominal | Living situation |
|  | INDEPEND | Ordinal | Level of independence |
|  | RESIDENC | Nominal | Type of residence |
|  | MARISTAT | Nominal | Marital status |
|  | HANDED | Nominal | Is the subject left- or right-handed? |
|  | TOBAC30 | Nominal | Smoked cigarettes in last 30 days |
| A5 Subject Health History | TOBAC100 | Nominal | Smoked more than 100 cigarettes in life |
|  | SMOKYRS | Ordinal | Total years smoked cigarettes |
|  | CVHATT | Ordinal | Heart attack/cardiac arrest |
|  | CVAFIB | Ordinal | Atrial fibrillation |
|  | CVANGIO | Ordinal | Angioplasty/endarterectomy/stent |
|  | CVBYPASS | Ordinal | Cardiac bypass procedure |
|  | CVPACE | Ordinal | Pacemaker |
|  | CVCHF | Ordinal | Congestive heart failure |
|  | CVOTHR | Ordinal | Other cardiovascular disease |
|  | CBSTROKE | Ordinal | Stroke |
|  | CBTIA | Ordinal | Transient ischemic attack (TIA) |
|  | SEIZURES | Ordinal | Seizures |
|  | DIABETES | Ordinal | Diabetes |
|  | TOBAC100 | Nominal | Smoked more than 100 cigarettes in life |
|  | DIABTYPE |  | If Recent/active or Remote/inactive Original UDS question v3 diabetes, which type? |
|  | HYPERTEN | Ordinal | Hypertension |
|  | HYPERCHO | Ordinal | Hypercholesterolemia |
|  | B12DEF | Ordinal | Vitamin B12 deficiency |
|  | THYROID | Ordinal | Thyroid disease |
|  | ALCOHOL | Ordinal | Alcohol abuse — clinically significant impairment occurring over a 12-month period manifested in one of the following areas: work, driving, legal, or social Numeric longitudinal |
|  | DEP2YRS | Nominal | Active depression in the last two years |
|  | DEPOTHR | Nominal | Depression episodes more than two years ago |
|  | PSYCDIS | Ordinal | Other psychiatric disorder |
|  | HEIGHT | Continuous | Subject’s height (inches) |
| B1 Physical | WEIGHT | Continuous | Subject’s weight (lbs) |
|  | BPSYS | Continuous | Subject blood pressure (sitting), systolic |
|  | BPDIAS | Continuous | Subject blood pressure (sitting), diastolic |
|  | HRATE | Continuous | Subject resting heart rate (pulse) |
|  | VISION | Nominal | Without corrective lenses, is the subject’s vision functionally normal? |
|  | HEARING | Nominal | Without a hearing aid(s), is the subject’s hearing functionally normal? |
| B4 Global Staging Clinical Dementia Rating (CDR) | MEMORY | Ordinal | Memory |
|  | ORIENT | Ordinal | Orientation |
|  | JUDGMENT | Ordinal | Judgment and problem-solving |
|  | COMMUN | Ordinal | Community affairs |
|  | HOMEHOBB | Ordinal | Home and hobbies |
|  | PERSCARE | Ordinal | Personal care |
|  | CDRSUM | Ordinal | Standard CDR sum of boxes |
|  | CDRLANG | Ordinal | Language |
|  | COMPORT | Ordinal | Behavior, comportment, and personality |
|  | NOGDS | Nominal | Is the subject able to complete the GDS, based on the clinician’s best judgment? |
| **B6 Geriatric Depression Scale (GDS)** | SATIS | Nominal | Are you basically satisfied with your life? |
|  | DROPACT | Nominal | Have you dropped many of your activities and interests? |
|  | EMPTY | Nominal | Do you feel that your life is empty? |
|  | BORED | Nominal | Do you often get bored? |
|  | SPIRITS | Nominal | Are you in good spirits most of the time? |
|  | AFRAID | Nominal | Are you afraid that something bad is going to happen to you? |
|  | HAPPY | Nominal | Do you feel happy most of the time? |
|  | HELPLESS | Nominal | Do you often feel helpless? |
|  | STAYHOME | Nominal | Do you prefer to stay at home, rather than going out and doing new things? |
|  | MEMPROB | Nominal | Do you feel you have more problems with memory than most? |
|  | WONDRFUL | Nominal | Do you think it is wonderful to be alive now? |
|  | WRTHLESS | Nominal | Do you feel pretty worthless the way you are now? |
|  | ENERGY | Nominal | Do you feel full of energy? |
|  | HOPELESS | Nominal | Do you feel that your situation is hopeless? |
|  | BETTER | Nominal | Do you think that most people are better off than you are? |
|  | NACCGDS | Ordinal | Total GDS Score |
| **B7 Functional Activities Questionnaire (FAQ)** | BILLS | Ordinal | In the past four weeks, did the subject have any difficulty or need help with: Writing checks, paying bills, or balancing a checkbook |
|  | TAXES | Ordinal | In the past four weeks, did the subject have any difficulty or need help with: Assembling tax records, business affairs, or other paper |
|  | SHOPPING | Ordinal | In the past four weeks, did the subject have any difficulty or need help with: Shopping alone for clothes, household necessities, or groceries |
|  | GAMES | Ordinal | In the past four weeks, did the subject have any difficulty or need help with: Playing a game of skill such as bridge or chess, working on a hobby |
|  | STOVE | Ordinal | In the past four weeks, did the subject have any difficulty or need help with: Heating water, making a |
|  | MEALPREP | Ordinal | In the past four weeks, did the subject have any difficulty or need help with: Preparing a balanced meal |
|  | EVENTS | Ordinal | In the past four weeks, did the subject have any difficulty or need help with: Keeping track of current events |
|  | PAYATTN | Ordinal | In the past four weeks, did the subject have any difficulty or need help with: Paying attention to and understanding a TV program, book, or magazine |
|  | REMDATES | Ordinal | In the past four weeks, did the subject have any difficulty or need help with: Remembering appointments, family occasions, holidays, medications |
|  | TRAVEL | Ordinal | In the past four weeks, did the subject have any difficulty or need help with: Traveling out of the neighborhood, driving, or arranging to take public transportation |
|  | NACCMMSE | Ordinal | Total Mini-Mental State Examination (MMSE) score (using D-L-R-O-W) |
